# Hypoxia-mediated translational activation of ITGB3 in breast cancer cells enhances TGF-β signaling and malignant features *in vitro* and *in vivo*

**DOI:** 10.18632/oncotarget.23145

**Published:** 2017-12-12

**Authors:** Marta Sesé, Pedro Fuentes, Anna Esteve-Codina, Eva Béjar, Kimberley McGrail, George Thomas, Trond Aasen, Santiago Ramón y Cajal

**Affiliations:** ^1^ Translational Molecular Pathology, Vall d’Hebron Research Institute, Universitat Autònoma de Barcelona, Barcelona, Spain; ^2^ Spanish Biomedical Research Network Centre in Oncology, CIBERONC, Barcelona, Spain; ^3^ CNAG-CRG, Centre for Genomic Regulation, Barcelona Institute of Science and Technology, Universitat Pompeu Fabra, Barcelona, Spain; ^4^ Biomedical Research in Melanoma-Animal Models and Cancer Laboratory, Oncology Program, Vall d'Hebron Research Institute, VHIR-Vall d'Hebron Hospital, Barcelona-UAB, Barcelona, Spain; ^5^ Division of Hematology/Oncology, Department of Internal Medicine, University of Cincinnati Medical School, Cincinnati, OH, USA; ^6^ Metabolism and Cancer Group, Molecular Mechanisms And Experimental Therapy In Oncology Program, Bellvitge Biomedical Research Institute, IDIBELL, Barcelona, Spain; ^7^ Physiological Sciences Department, Faculty of Medicine and Health Science, University of Barcelona, Barcelona, Spain

**Keywords:** hypoxia, polysomes, breast cancer, migration, integrin beta 3

## Abstract

Breast cancer is the most prevalent malignancy in women and there is an urgent need for new therapeutic drugs targeting aggressive and metastatic subtypes, such as hormone-refractory triple-negative breast cancer (TNBC). Control of protein synthesis is vital to cell growth and tumour progression and permits increased resistance to therapy and cellular stress. Hypoxic cancer cells attain invasive and metastatic properties and chemotherapy resistance, but the regulation and role of protein synthesis in this setting is poorly understood. We performed a polysomal RNA-Seq screen in non-malignant breast epithelial (MCF10A) and TNBC (MDA-MB-231) cells exposed to normoxic or hypoxic conditions and/or treated with an mTOR pathway inhibitor. Analysis of both the transcriptome and the translatome identified mRNA transcripts translationally activated or repressed by hypoxia in an mTOR-dependent or -independent manner. Integrin beta 3 (ITGB3) was translationally activated in hypoxia and its knockdown increased apoptosis and reduced survival and migration, particularly under hypoxic conditions. Moreover, ITGB3 was required for sustained TGF-β pathway activation and for the induction of Snail and associated epithelial-mesenchymal transition markers. ITGB3 downregulation significantly reduced lung metastasis and improved overall survival in mice. Collectively, these data suggest that ITGB3 is translationally activated in hypoxia and regulates malignant features, including epithelial-mesenchymal transition and cell migration, through the TGF-β pathway, revealing a novel angle for the treatment of therapy-resistant hypoxic tumours.

## INTRODUCTION

Breast cancer is the most common malignancy and the second leading cause of cancer-related death in adult women [1]. Despite significant advances in the characterization of breast cancer subtypes and the development of new therapeutic approaches, advanced and aggressive forms of breast cancer continue to have a poor prognosis [2]. Triple-negative breast cancer (TNBC) is an important subtype of breast cancer defined by the loss of estrogen receptor, progesterone receptor and human epidermal growth factor receptor type 2 (HER2), all of which are clinically important therapeutic targets because the major therapeutic strategies in breast cancer are hormone therapy and/or HER2 antibodies [3]. TNBC is a highly aggressive tumour subtype with high risk of recurrence, metastasis, chemotherapy resistance and acquired capacity to survive and grow under nutrient-deprived and hypoxic (low-oxygen) conditions. TNBC also appears to adopt a unique response to cell stress by mimicking a hypoxia gene signature associated with poor prognosis [4]. Under hypoxic conditions, TNBC cells can grow, survive, induce metabolic reprogramming and apoptosis and alter cell adhesion and motility to facilitate metastasis and resistance to chemotherapy [5, 6]. Most of these phenotypes involve several transcriptional changes mainly related to the stabilization of the master transcription factor HIF1α [7, 8]. However, little is known about mRNA regulation at the translational level under low-oxygen conditions [9, 10], although prolonged exposure to hypoxia inhibits translation via repression of the mTOR (mechanistic target of rapamycin) signalling pathway more efficiently at 0.3%–0.5% O_2_ compared to 1% of O_2_ concentration [10, 11]. Little is known about translational regulated targets in low oxygen conditions, in particular those subsets of mRNAs that are still capable to be translated that may favour cell migration and survival of TNBC cells under this stress.

Tumours display a high rate of protein synthesis [12] and several studies have shown that control of mRNA translation is critical not only for survival under hypoxic conditions, but also for cancer initiation, progression, migration and invasion [13, 14]. Translation is mostly controlled at initiation, when eukaryotic translation initiation factors (eIFs) are recruited to the 5′-m7G cap structure of mRNA, forming the eIF4F complex that recruits the eukaryotic small 40S ribosomal subunit. This step is mainly regulated by the mTOR signalling pathway [15–17]. mTOR forms two distinct complexes, mTOR complex 1 (mTORC1) and mTOR complex 2 (mTORC2) [18]. mTORC1 is composed of mTOR, regulatory-associated protein of mTOR (Raptor), mammalian LST8/G-protein β-subunit-like protein (mLST8/GβL) and the PRAS40 and DEPTOR partners [19] and is a master regulator of protein synthesis that couples nutrient sensing to cell growth and cancer cell survival. The major regulators of protein synthesis downstream of mTORC1 are 4E-BP1 and p70S6K1/2 [19–21]. Upon phosphorylation by mTORC1, 4E-BP1 releases eIF4E, which can then recruit eIF4A and eIF4G to form the eIF4F complex [22, 23]. This global downregulation of protein synthesis under low-oxygen conditions coincides with a simultaneous enhanced translation of certain mRNAs that encode proteins involved in adaptation to cellular stress. Activation of the eIF4F initiation complex is compromised under hypoxic conditions [9, 24] in favour of an eIF4F^H^ complex composed of eIF4E2, eIF4A and eIF4G3 [25]. HIF2α is induced by hypoxia and binds to a specific element in the 3′UTR (untranslated region) of some mRNA transcripts, allowing their selective translation initiation via recruitment of eIF4E2 [25–27]. Transcripts activated through this mechanism include the epidermal growth factor receptor (EGFR) and the insulin growth factor receptor (IGFR1) [26].

Another proposed mechanism to ensure protein synthesis when cap-dependent translation is inhibited is IRES (internal ribosome entry site)-mediated translation [28–30]. The presence of potential IRES elements in the 5′UTR of certain mRNAs such as HIF1α, VEGF and c-MYC enables activation of their translation through a cap-independent pathway in hypoxia.

Finally, a third mechanism is the observation that, in hypoxia, uORF (upstream ORF)-mediated translation is enhanced due to an increase in eIF2α phosphorylation by PERK. Transcripts containing uORFs in their 5′UTR include genes related to proliferation and cell survival under stress conditions such as ATF4, CHOP and GADD34 [31].

Polysome profiling is a standardized technique to capture mRNA translation by immobilizing actively translating mRNAs on ribosomes and separate the resulting polyribosomes by ultracentrifugation on a sucrose gradient, thus allowing for an analysis of translated mRNAs compared to total mRNA [32]. The combined use of polysomal fractionation with microarray analysis or of ribosome profiling with high-throughput RNA sequencing (RNA-Seq) has identified specific transcripts actively translated under low-oxygen conditions in tumour cells [24, 25, 33–35]. These approaches have already revealed the ability of an mTORC1 inhibitor to block cap-dependent translation in the translatome of both fibroblasts and PC-3 cells [36, 37]. Nevertheless, the mechanisms underlying this selective translation are poorly understood, as well as how it affects the behaviour and survival of cancer cells exposed to hypoxia.

ITGB3 is an integrin that forms heterodimers with alpha chains, either ITGAV or ITGAIIb. These adhesion molecules are receptors for fibronectin, vitronectin, collagen and laminin and facilitate attachment between the cell cytoskeleton and the extracellular matrix [38]. Most integrins induce transmembrane signalling through activation of focal adhesion kinase (FAK) and Src family kinases that in turn activate downstream effectors such as small GTPases. Functional inhibition of ITGB3 suppresses neovascularisation, tumour growth and metastasis, suggesting that αvβ3 integrin may be a critical modulator of pathological angiogenesis [39–44]. ITGB3 is expressed in a subpopulation of breast cancer stem cells and is associated with poor outcome [45, 46].

In this study, we used sucrose gradient fractionation and polysome profiling to separate and quantify actively translating mRNAs bound to ribosomes. We sequenced total and polysomal mRNAs and examined the effects of hypoxia alone and hypoxia combined with mTOR inhibition to avoid standard cap-dependent translation on the translatome of the non-malignant MCF10A epithelial breast cell line and the triple-negative MDA-MB-231 breast cancer cell line. Our screen identified translational regulation of a number of genes under highly inhibitory and stressful situations, including ITGB3, which was upregulated at the protein synthesis level in hypoxia. Increased ITGB3 facilitates the migratory and invasive capabilities of breast cancer cells through induction of Snail and epithelial-mesenchymal transition (EMT) via the TGF-β pathway, an effect particularly evident under hypoxic conditions. Moreover, ITGB3 silencing with shRNA reduced tumourigenesis *in vitro* and *in vivo*, suggesting that ITGB3 is a candidate therapeutic target for most aggressive breast tumours able to survive under low-oxygen conditions.

## RESULTS

### Combination of hypoxia and mTOR inhibitor treatment identifies a unique subset of genes regulated at both transcriptional and translational levels

**Figure 1:**
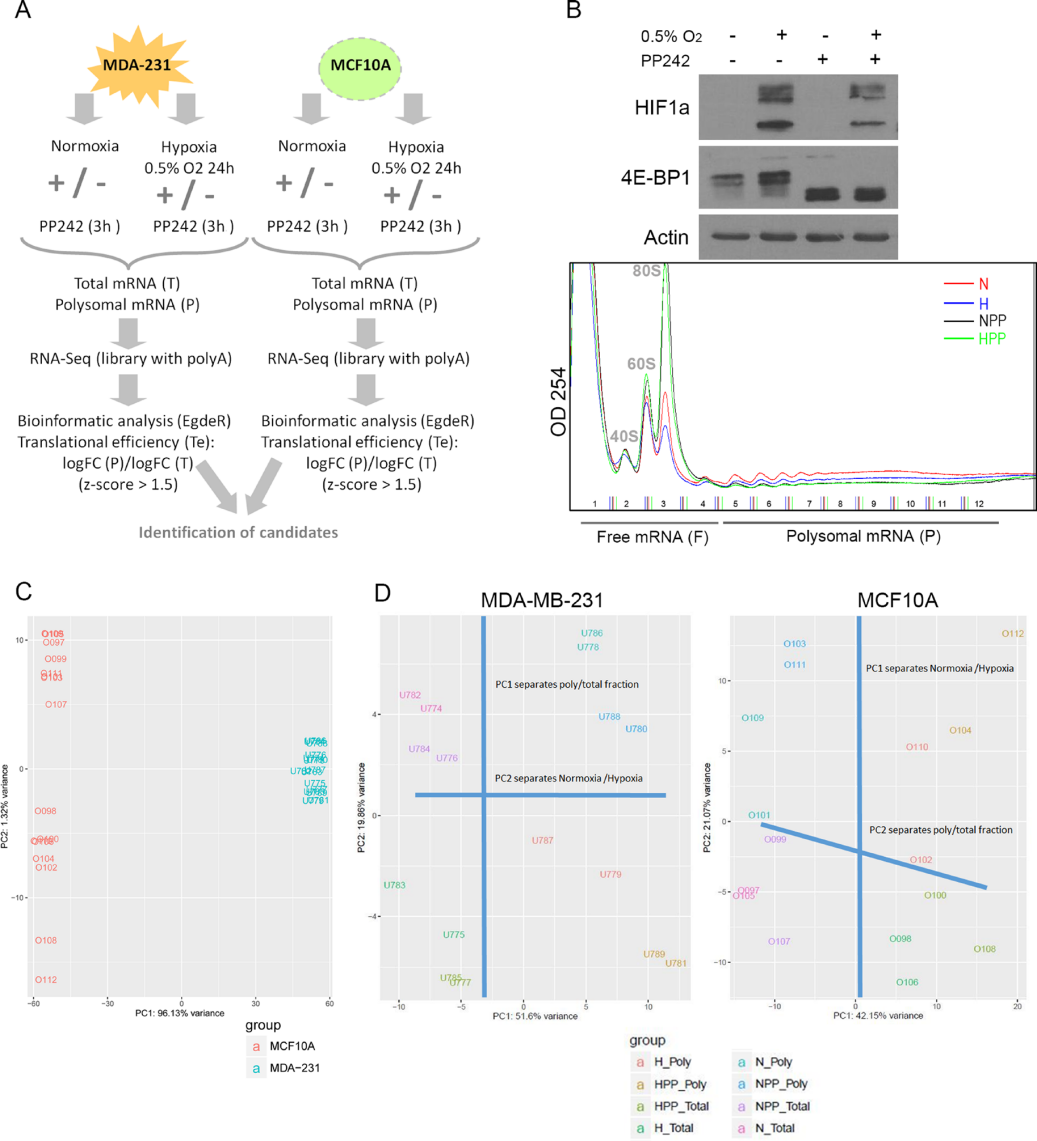
Overview of the polysomal RNA-Seq screen after hypoxia and mTOR inhibition (**A**) Schematic workflow of the experiment. (**B**) Above: Immunoblot of HIF1α and 4E-BP1 under normoxic (N), hypoxic (H), PP242 (PP) and hypoxic + PP242 (HPP) conditions. Below: Polysome profiles of MCF10A cells in all conditions. (**C**) PCA plot of all samples of the dataset to emphasize the variation between replicates, treatments and cell lines. The first component (PC1) explained 96% of the total variance while PC2 explained 32%. (**D**) PCA plot of MDA-MB-231 (left) and MCF10A (right) samples showing that one PC separates total from polysomal mRNA and the other separates normoxic from hypoxic conditions.

We aimed to analyse differential transcription and translation efficiencies under hypoxic conditions by comparing non-tumourigenic cells (MCF10A) to malignant TNBC cells (MDA-MB-231). We conducted a polysomal RNA-Seq screen after exposing cells to 24 hours of hypoxia (0.5% O_2_) or normoxia (21% O_2_), with or without 3-hour treatment with the mTORC1 and -2 inhibitor PP242 (Figure 1A). PP242 treatment was used to discriminate between mRNAs that were still bound to polysomes after mTORC1/−2 inhibition under low-oxygen conditions in order to identify transcripts supposedly translated in a cap-independent manner. As expected, HIF1α accumulated in cells exposed to hypoxia, and its induction was slightly diminished in cells co-treated with PP242 [47], which dephosphorylated 4E-BP1 (Figure 1B) and thereby inhibited cap-dependent translation. Under all four conditions, total mRNA was isolated and fractionated using a sucrose gradient to separate monosomes and oligosomes (free mRNA, F) from the actively translated mRNAs bound to polysomes (polysomal mRNA, P) (Figure 1B). A systematic analysis of the total (T) and polysome-bound (P) mRNA of each sample was carried out by high-throughput RNA-Seq and analysed bioinformatically to obtain differential gene expression with regards to both the transcriptome and translatome in the two cell lines.

Principal component analysis (PCA) of the raw data clearly separated MCF10A and MDA-MB-231 samples in accordance with the highly different transcriptomes of the two cell types (Figure 1C). The within-cell type variability upon treatment, which is reflected by principal component 2 (PC2), appeared to be higher for the MCF10A samples than the tumour samples (Figure 1C), meaning that non-tumourigenic MCF10A cells were more affected by hypoxia or PP242 than tumoural MDA-MB-231 cells. Independent PCAs were performed for each cell type dataset to inspect in greater detail the clustering within each cell type. In tumour cells, PC1 mainly reflected variance attributable to differences between the total mRNA and polysome-bound mRNA and PC2 differentiated normoxic and hypoxic conditions. In non-tumoural cells, the pattern was the reverse (Figure 1D). However, in both cases, PC3 explained the drug effect (PP242 vs non-PP242) (data not shown). These results confirmed that there was variability between cell lines and within treatments.

**Figure 2:**
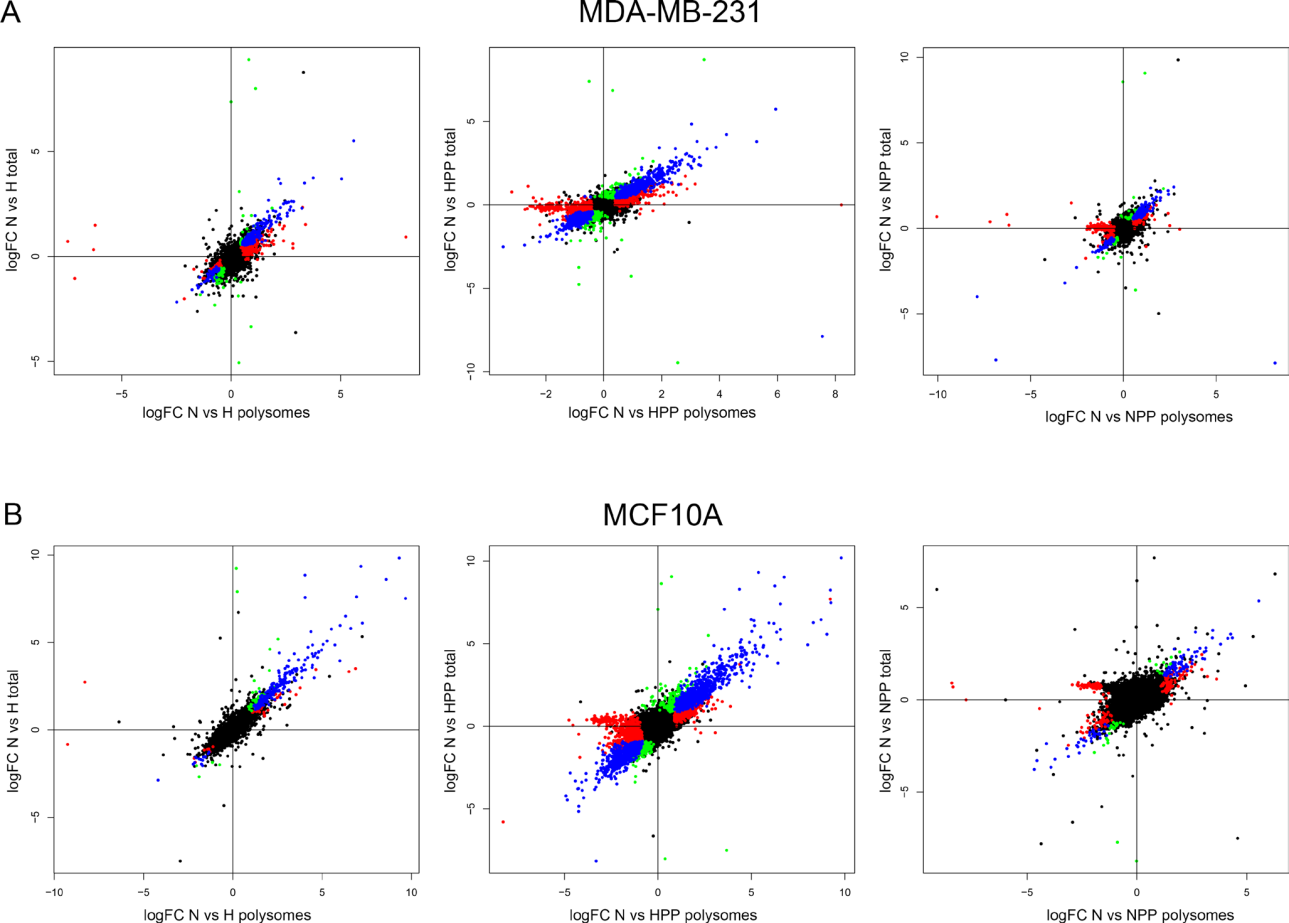
Transcriptional and translational changes in non-tumoural and malignant cells under hypoxic and mTOR inhibition conditions (**A** and **B**) Correlation between transcript expression in total mRNA versus mRNA in polysomal fractions under the different conditions in (A) MCF10A and (B) MDA-MB-231 cell lines. Classification of the transcripts as (I) deregulated at the transcriptional level (blue), (II) translationally activated or inhibited (red) and (III) not significantly regulated (black). Overall, the results show that more genes are regulated transcriptionally and translationally in non-tumoural cells than in tumoural cells.

**Figure 3:**
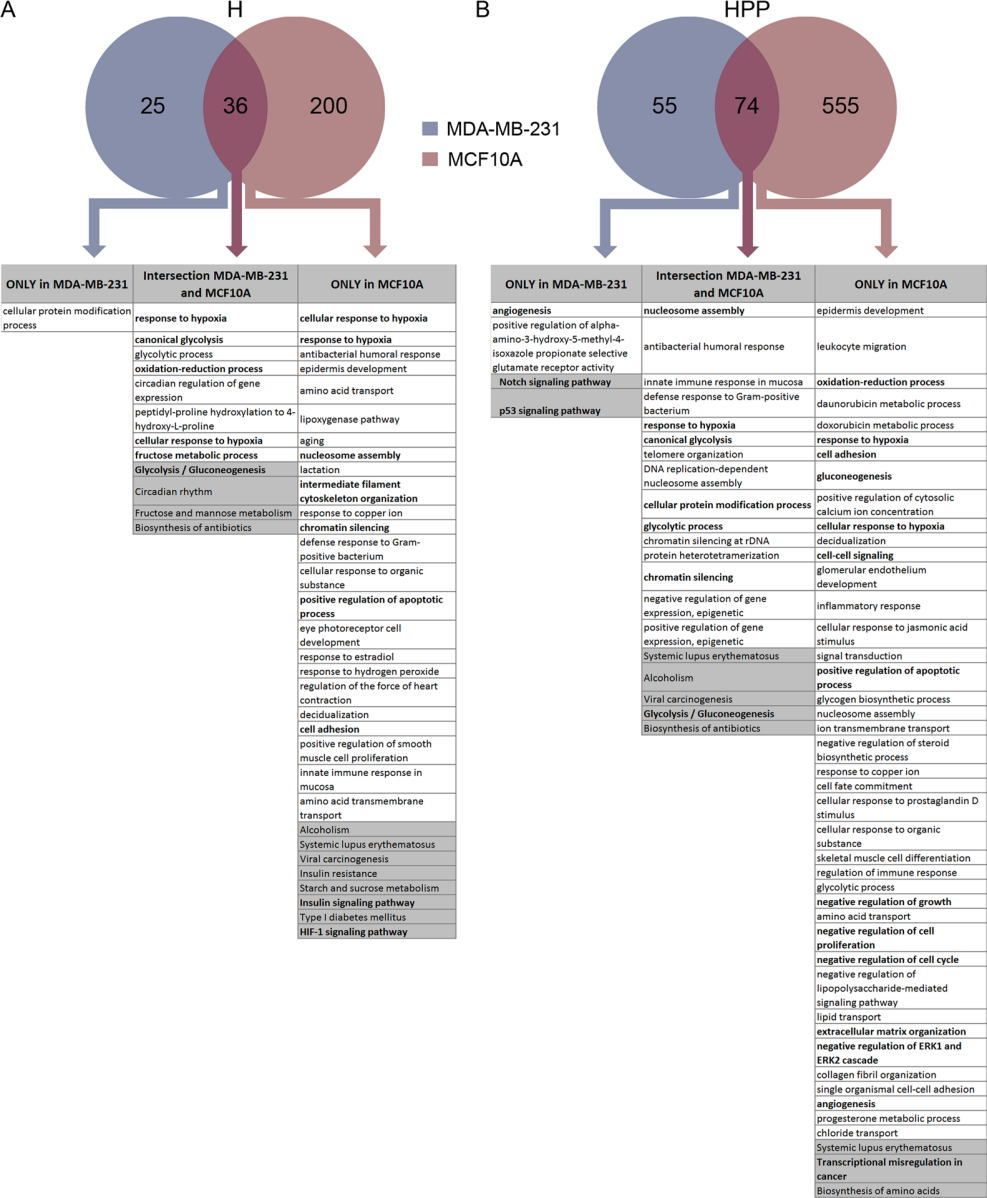
Transcriptome analysis of MCF10A and MDA-MB-231 cells after hypoxia and hypoxia + PP242 (**A**) Venn diagram of upregulated transcripts in hypoxia. GO (in white boxes, with *P* < 0.03) and Kegg pathway analysis (in grey boxes, with *P* < 0.1) of gene sets enriched only in MCF10A cells, only in MDA-MB-231 cells and in the intersection between these two cell lines. (**B**) Venn diagram of upregulated transcripts in hypoxia + PP242. GO (in white boxes, with *P* < 0.03) and Kegg pathway analysis (in grey boxes, with *P* < 0.1) of gene sets enriched only in MCF10A cells, only in MDA-MB-231 cells and in the intersection between these two cell lines.

Following bioinformatic analysis of the differential gene expression in both total (T) and polysome-bound (P) mRNA, the fold change expression (log_2_FC) levels were calculated by comparing all conditions with the control condition of normoxia and plotting the correlation between log_2_FC_P and log_2_FC_T for each treatment. There was a close correlation between differentially expressed genes in total mRNA versus genes differentially expressed in polysomes, meaning that major transcriptional changes were also reflected at the level of translation (blue colour in Figure 2). We classified the genes into four groups: (1) transcriptionally upregulated or downregulated (blue colour in Figure 2); (2) transcriptionally upregulated or downregulated but no changes in translation (green colour in Figure 2); (3) translationally upregulated or downregulated (red colour in Figure 2); and (4) non-significant changes (black colour in Figure 2). Notably, the translation of some genes was specifically activated or inactivated by the different treatments (red colour in Figure 2). In general, we observed the most significant activation and inhibition, both transcriptionally and translationally, upon double hypoxia + PP242 treatment. Normoxia + PP242 treatment alone did not affect transcription, but potently inhibited translation, as expected. Moreover, in all conditions, MCF10A cell lines were more affected at the level of both transcription and translation than MDA-MB-231 cells, supporting the findings of our PCA analysis (Figure 1C). These findings mean that MCF10A cells have to enact major changes to survive under low-oxygen conditions (Figure 2).

### Transcriptional changes are more extensive in the non-tumourigenic cell line in both hypoxia and combined hypoxia + PP242

Next, we analysed the transcriptional differences between the two cell lines upon hypoxia (H) and hypoxia + PP242 (HPP) treatment. We detected 236 upregulated and 17 downregulated genes in MCF10A cells and 61 upregulated and 9 downregulated genes in MDA-MB-231 cells at the transcriptional level in hypoxia; 23 of these genes were the same in the two cell lines (Supplementary Figure 1). By analysing the function of genes upregulated in the intersection between the two cell lines, we found that most of the Gene Ontology (GO) categories were related to response to hypoxia and glycolysis and oxidation-reduction processes, as expected (Figure 3A). The MDA-MB-231 cell line did not show any significant GO-enriched category (perhaps because fewer genes were applied to the analysis), whereas the genes upregulated in MCF10A cells were associated with nucleosome assembly, apoptosis, angiogenesis and proliferation. This difference may suggest that changes induced by hypoxia in genes associated with malignant features are more extensive in the non-tumourigenic cell line than in the tumourigenic TNBC cell line.

Transcriptional changes were more evident when cells were treated with combined hypoxia + PP242, especially in MCF10A cells, which showed more up- and downregulated transcripts than MDA-MB-231 cells (Supplementary Figure 1). In particular, 631 mRNAs were upregulated in MCF10A cells upon HPP treatment, compared with only 130 genes in MDA-MB-231 cells, with 74 genes common to the two cell lines. Again, GO analysis indicated that the genes in the intersection were devoted to the response to hypoxia, nucleosome assembly and glycolysis categories. In cancer cells, angiogenesis and the Notch signalling and p53 pathways were upregulated. In MCF10A cells, cell adhesion, cell–cell signalling, apoptosis, growth, proliferation and cell cycle categories were upregulated, indicating a more organized change in the non-tumourigenic cell line towards a full EMT program (Figure 3B). On the other hand, genes transcriptionally downregulated under H and HPP conditions were mainly related to cell proliferation and cell cycle in the two cell lines (Supplementary Figure 2A). In terms of GO categories and pathways downregulated in HPP, minor changes were observed in MDA-MB-231 cells. However, in MCF10A cells, several signalling pathways were downregulated, such as the Wnt pathway, the Hippo pathway, the TGF-β pathway and pathways related to the cell cycle (Supplementary Figure 2B). As expected, no significant transcriptional changes were observed in cells treated with PP242 alone (Supplementary Figure 3). Genes transcriptionally deregulated in each condition are listed in Supplementary Table 1.

Although many of the genes transcriptionally upregulated upon hypoxia + PP242 treatment are important for cell survival, we focused our attention on genes activated at the protein synthesis level, a less understood and studied feature.

### The MCF10A and MDA-MB-231 translatome in hypoxia and hypoxia + PP242

**Figure 4:**
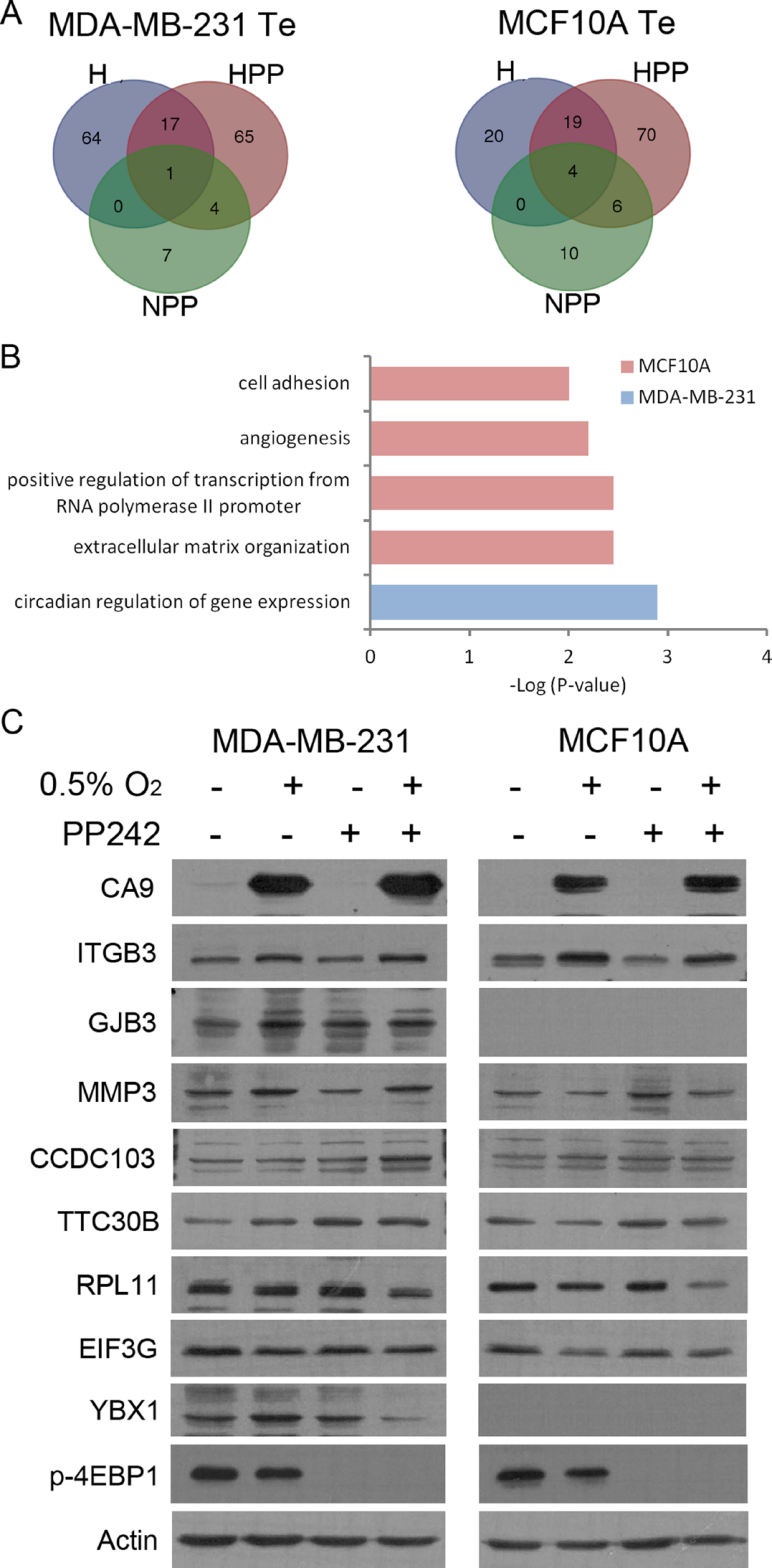
Increased translational efficiency (Te) is accompanied by increased protein (**A**) Venn diagram of Te distribution of transcripts in MDA-MB-231 (left) and MCF10A (right) cells under all three experimental conditions. (**B**) GO analysis of Te under HPP conditions in both cell lines. (**C**) Immunoblots for all experimental conditions of the different translationally activated (ITGB3, GJB3, MMP3, CCDC103, TTC30B) or inactivated (RPL11, EIF3G, YBX1) targets in HPP.

We analysed the translational efficiency (Te) to identify translationally activated (*z*-score > 1.5) or inactivated (*z*-score < 1.5) genes under H, HPP and NPP conditions (Supplementary Table 1). In hypoxia, 82 and 43 genes were upregulated and 12 and 22 genes were downregulated at the translational level in MDA-MB-231 and MCF10A cells, respectively (Figure 4A, Supplementary Figure 4B, Supplementary Table 1). This suggests that a limited number of genes are significantly regulated at the level of translation under hypoxic conditions. Curiously, more genes were regulated at the translational level in the tumour cell line (MDA-MB-231) than in non-tumoural cells (MCF10A), which is the opposite of what was occurring in terms of transcriptional changes. With hypoxia + PP242 treatment, we detected 87 and 99 genes translationally upregulated and 224 and 450 translationally inactivated in MDA-MB-231 and MCF10A cells, respectively (Figure 4A, Supplementary Figure 4B, Supplementary Table 1). However, as expected, mRNAs were mainly translationally inactivated in HPP and NPP upon mTOR inhibitor treatment. Twice as many genes were downregulated in tumoural cells, possibly due to a highly activated mTOR pathway in this cell line, which naturally increases the number of effective PP242 targets. GO categories of activated transcripts indicated that the genes were mainly related to cell adhesion, angiogenesis and extracellular matrix organization in MCF10A cells (Figure 4B), whereas the only significant GO category in the cancer cell line was circadian regulation of gene expression.

Genes inactivated by H and HPP in both cell lines are presented in Supplementary Figure 4 and were mainly related to translation, catabolic processes and mitochondrial transport in both cell lines (Supplementary Figure 4A–4C). Genes translationally activated/inactivated in each condition are listed in Supplementary Table 1.

As a validation of our screening, most of the genes inactivated under NPP conditions, of which nearly 50% were common to the two cell lines, were cap-dependent genes containing TOP sequences. These *cis*-acting elements are found in some mRNAs that are mostly localized to polysomes in actively growing cells whose translational activation is mainly regulated by the mTOR signalling pathway [48, 49], and most were the same as those described by Thoreen and colleagues and Hsieh and colleagues after mTOR inhibition of other cell lines [36, 37] (Supplementary Figure 4D). To further validate our screening at the protein level, candidate genes that were translationally activated or inactivated in HPP were analysed by western blotting. Candidate genes with a Te higher than 1.5 displayed an increase at the protein level under HPP conditions compared with control in both MCF10A cells (TTC30B and ITGB3) and MDA-MB-231 cells (CCDC103, ITGB3, Cx31, MMP3 and TTC30B). Although we picked these candidates from genes translationally activated by HPP, in most cases, we also observed more protein under hypoxic conditions alone. We detected reduced expression when analysing proteins from translationally inactivated genes such as RPL11, EIF3G and YBX1 (Figure 4C). As expected, we also detected increased protein expression of HIF1α target genes such as CA9 in hypoxia (Figure 4C). These set of proteins activated / intactivated translationally were also validated in other breast cancer cell lines such as MCF7, MDA-MB-468 and BT-549 (Supplementary Figure 5) to ensure that this phenomena was occurring in a more general way. Importantly, we observed that ITGB3 was overexpressed in H and HPP in all cell lines analyzed.

The screening was also validated by quantitative real time RT-PCR (qRT-PCR) for some targets using the 50-gene PAM50 assay (Prosigna; NanoString Technologies, Seattle, WA) [50, 51], which is designed to identify clinically relevant molecular subtypes of breast cancer [52]. We observed a strong correlation between our RNA-Seq results and our NanoString analysis (Supplementary Figures 6 and 7).

Altogether, our RNA-Seq analysis showed that transcripts found to be transcriptionally and translationally activated in hypoxia or in hypoxia + PP242 were also found to be increased with qRT-PCR and NanoString and at the protein level by western blot, which validates our multi-level screening approach and suggests that some of the identified genes may be relevant in hypoxia, for example, by promoting survival and/or migration capabilities.

### ITGB3 is translationally activated in hypoxia and hypoxia + PP242 and promotes cell migration in hypoxia *in vitro* and metastasis establishment *in vivo*

A secondary functional screening was performed using siRNA from the list of candidate genes actively translated (Te > 1.5) in both cell lines. Both proliferation and migration assays were conducted (Supplementary Figure 8). Of the candidates, ITGB3 silencing in MDA-MB-231 cells significantly reduced cell viability specifically in hypoxia and not in normoxia (Supplementary Figure 8A and 8B). Moreover, increased apoptosis was observed in hypoxia but not normoxia upon ITGB3 silencing (Supplementary Figure 8D). In contrast, there was a tendency for decreased cell migration (Supplementary Figure 8C).

**Figure 5:**
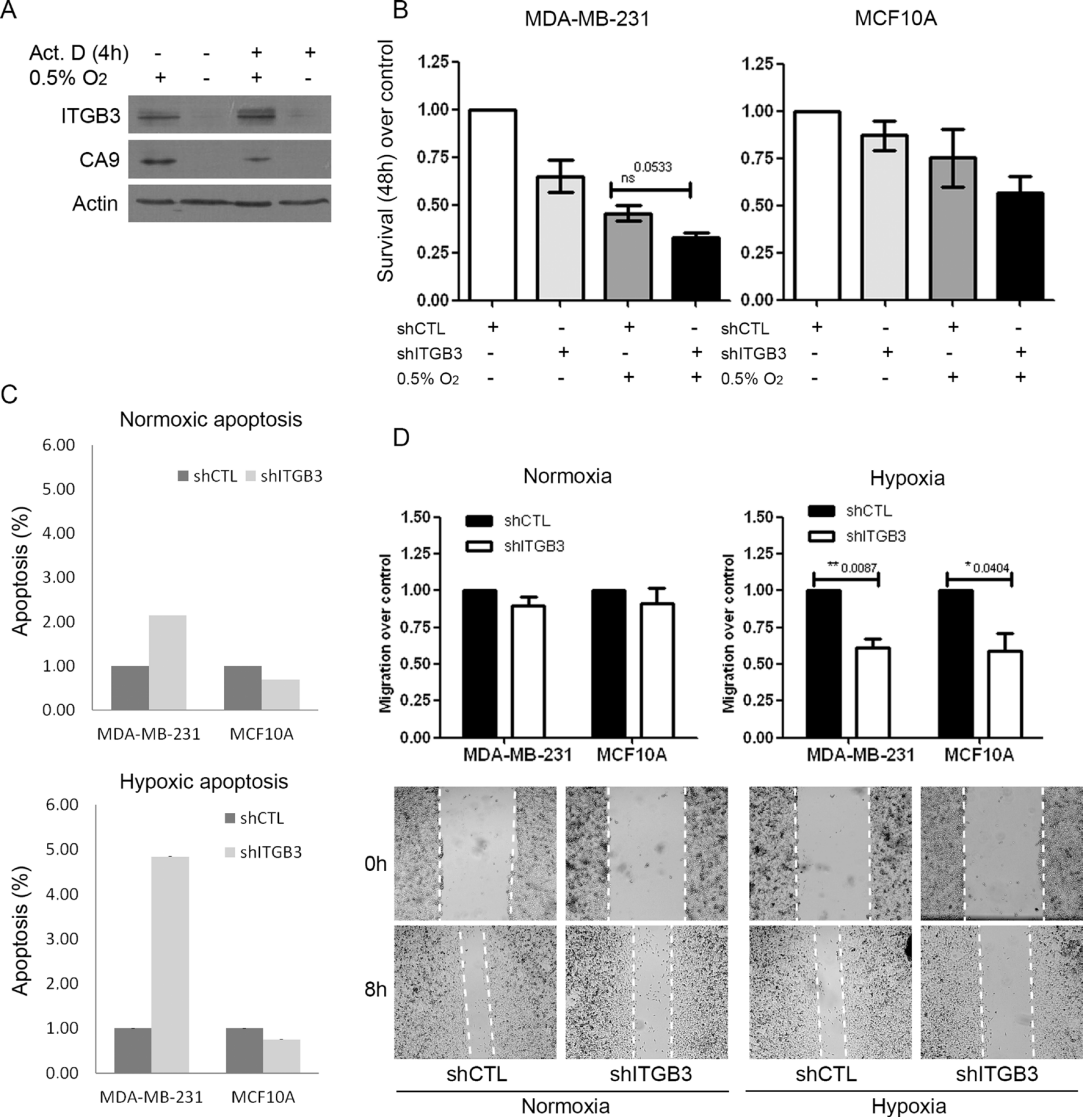
ITGB3 is translationally activated under hypoxic conditions and is important for breast cell line survival and migration (**A**) Immunoblots of ITGB3 in MDA-MB-231 cells subjected to hypoxia or normoxia and treated with actinomycin D. (**B**–**D**) Phenotypes of stably shITGB3-infected cells. Effects of ITGB3 depletion on (B) survival visualized by MTT assay at 48 hours, (C) apoptosis using caspase-3/−7 activation and (D) migration in a wound healing assay in hypoxia versus normoxia.

Notably, when analysing GO categories of translationally activated genes in HPP in MCF10A cells, we observed a general activation of integrins at the protein synthesis level, particularly ITGB3, ITGB4, ITGAX and ITGA5. Based on this finding and the results of our preliminary siRNA screen, we therefore performed a more detailed analysis of ITGB3 and its regulation and role in hypoxia. Considering the important role of ITGB3 in tumourigenesis, we further studied ITGB3 in breast cancer cells under hypoxic conditions. To further validate the role of ITGB3, cells were treated with actinomycin D to avoid transcriptional changes and were subjected to hypoxia. ITGB3 protein was still induced in hypoxia in the absence of mRNA synthesis (Figure 5A), in concordance with our RNA-Seq data, suggesting that this is a translational rather than transcriptional event. As a control, we studied CA9, a well-known effector of HIF1α, which was not activated in hypoxia upon treatment with actinomycin D (Figure 5A).

Having validated that ITGB3 was upregulated at the protein level in HPP and also under hypoxic conditions alone, we explored putative functional roles of ITGB3 in breast cancer progression, particularly under low-oxygen conditions. Similar to our siRNA screen (Supplementary Figure 8C), knockdown of ITGB3 using viral shRNA approaches reduced cell proliferation under both normoxic and hypoxic conditions in MCF10A and MDA-MB-231 cells (Figure 5B). Furthermore, ITGB3 depletion induced apoptosis, with more evident effects in hypoxia (Figure 5C and Supplementary Figure 8D). Knockdown of ITGB3 significantly decreased cell migration, but this was more prominent and significant under low-oxygen conditions in both cell lines (Figure 5D). This suggests that ITGB3 is important for cell migration and survival in both cancer and non-malignant cells, but particularly under hypoxic conditions.

**Figure 6:**
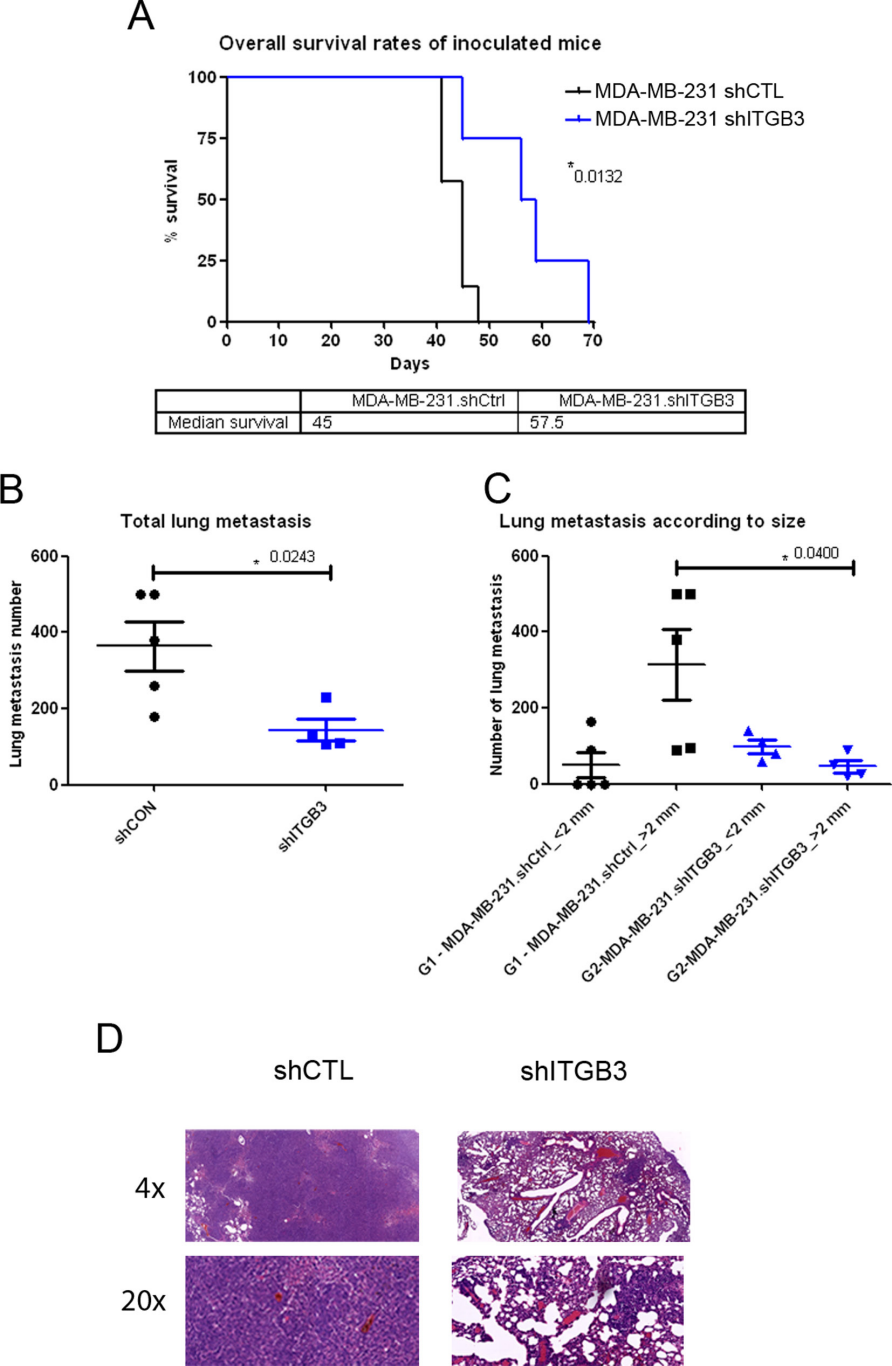
Survival and lung metastasis after intravenous inoculation with ITGB3-depleted MDA-MB-231 human breast cancer cells (**A**) Overall survival rates of inoculated mice. Downregulation of ITGB3 protein significantly increased the overall survival rate of mice inoculated with the MDA-MB-231.shITGB3 cell variant. Median survival times were 45.0 days and 57.5 days for the MDA-MB-231.shCtrl- and MDA-MB-231.shITGB3-inoculated groups, respectively. Subsequently, the two Kaplan-Meier curves and estimates of survival showed them to be significantly different (*P* = 0.0132). (**B** and **C**) Comparative analysis of the lung metastasis number (B) and number per size (C) of MDA-MB-231.shCtrl- and MDA-MB-231.shITGB3-inoculated groups at the end time point. Lines indicate the median corresponding values of the groups. Downregulation of ITGB3 protein decreased lung metastasis growth of breast cancer with respect to control animals, with significant differences in lung total number (*P* = 0.0213) (B) and in number per size (*P* = 0.0400) (C). (**D**) Hematoxylin and eosin staining of mouse lung sections were tumours of shCTL and shITGB3 MDA-MB-231 cell lines are evident.

Next, we assessed ITGB3 function *in vivo* by injecting control and ITGB3-silenced cells into the mouse tail. Our results suggested that cancer cells with silenced ITGB3 form fewer metastases and those that do appear are smaller than with control non-silenced tumour cells (Figure 6B–6D). This was reflected in the improved overall survival of animals injected with ITGB3-silenced MDA-MB-231 cells compared with non-silenced cells (Figure 6A).

### ITGB3 amplifies TGF-β signalling in hypoxia, enhancing the EMT and cell migration

**Figure 7:**
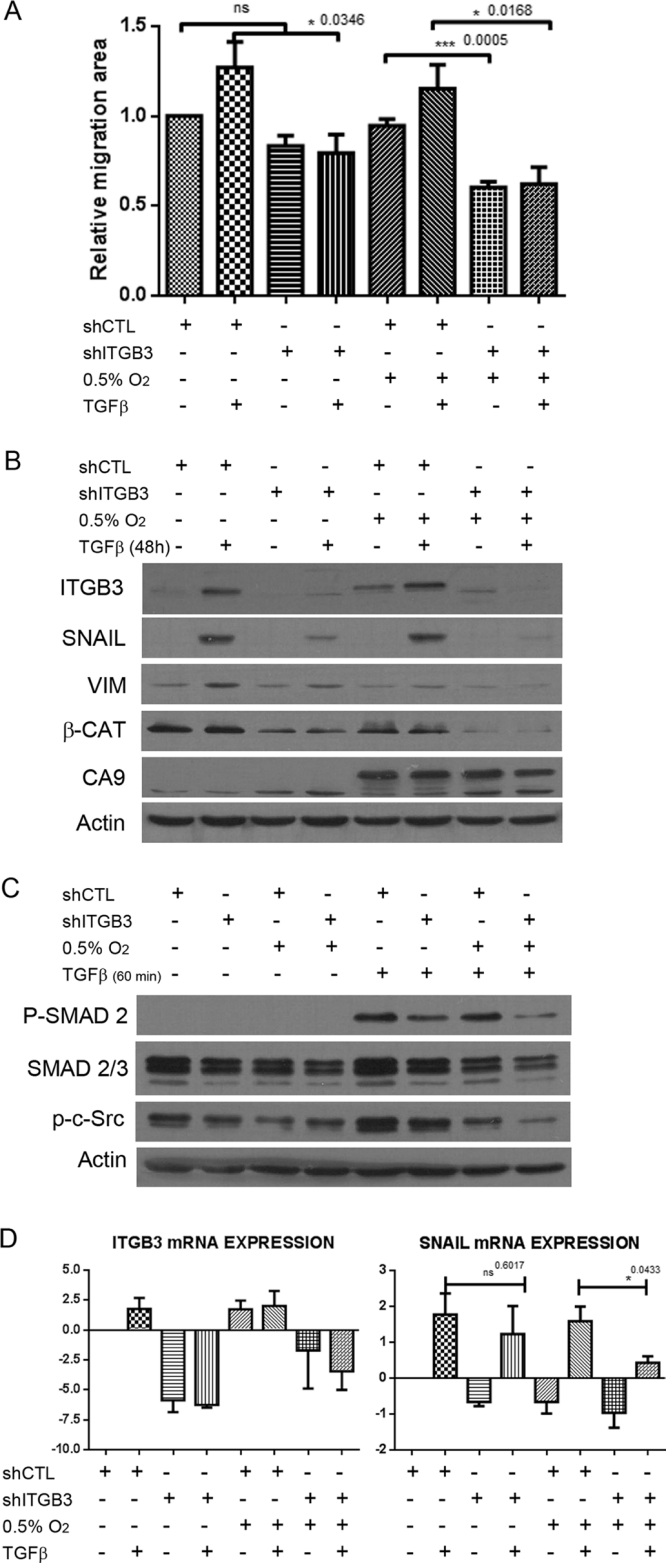
ITGB3 depletion blocks TGF-β pathway activation more efficiently in hypoxia than in normoxia (**A**) Migration assays of MDA-MB-231.shITGB3 cells in hypoxia versus normoxia and treated with TGF-β. (**B**) Immunoblot of MDA-MB-231.shITGB3 and MDA-MB-231.shCTL cells treated with TGF-β and subjected to hypoxia or normoxia for 48 hours to analyse the expression of EMT factors such as Snail and vimentin. (**C**) Immunoblot analysis of Smad2 phosphorylation in MDA-MB-231 cells infected with shITGB3 or control shCTL subjected to hypoxia or normoxia for 24 hours and treated with TGF-β for 60 minutes. (**D**) qRT-PCR of cells treated with TGF-β and subjected to hypoxia or normoxia for 48 hours to analyse the expression of EMT factors such as Snail and vimentin.

Because ITGB3 interacts with TGF-β receptor II in mammary epithelial cells and enhances its function through Src [53], we wondered whether the TGF-β pathway was modulated by ITGB3 under hypoxic conditions. We measured cell migration in control and ITGB3-silenced MDA-MB-231 cells with or without treatment with TGF-β, a well-known stimulator of EMT and migration in cancer cells [54]. As expected, TGF-β increased the rate of cell migration in both normoxia and hypoxia (Figure 7A). However, when ITGB3 was silenced, TGF-β treatment failed to induce and even partially reduced cell migration, especially under hypoxic conditions. Accordingly, TGF-β increased ITGB3 expression in control cells but not in shITGB3 cell lines. Moreover, increased expression of the important EMT-associated transcription factor Snail was observed at the mRNA and protein levels after TGF-β treatment in control cells but not when ITGB3 was silenced. In fact, we observed a clear downregulation of Snail expression upon ITGB3 silencing, which was especially evident and significant in hypoxia (Figure 7B and 7C). The same observations were made using MCF10A cells, indicating that this integrin is required for proper TGF-β signalling and regulation of Snail (Supplementary Figure 9). These results clearly suggest that ITGB3 is required for TGF-β-mediated expression of Snail, particularly under low-oxygen conditions. In support, reduced TGF-β-mediated induction of Snail-regulated EMT genes such as vimentin and N-cadherin was observed in ITGB3-silenced cells (Figure 7B and Supplementary Figure 9). Finally, we observed maximum phosphorylation of Smad2 after 1-hour treatment. At this time point, Smad was not phosphorylated when cells silenced with ITGB3 (Figure 7D). These findings suggest that ITGB3 affects the classical TGF-β signalling pathway at an early stage, possibly by directly acting on the receptor itself, as previously suggested [53].

### EIF4E is essential for the translational activation of ITGB3 in hypoxia

**Figure 8:**
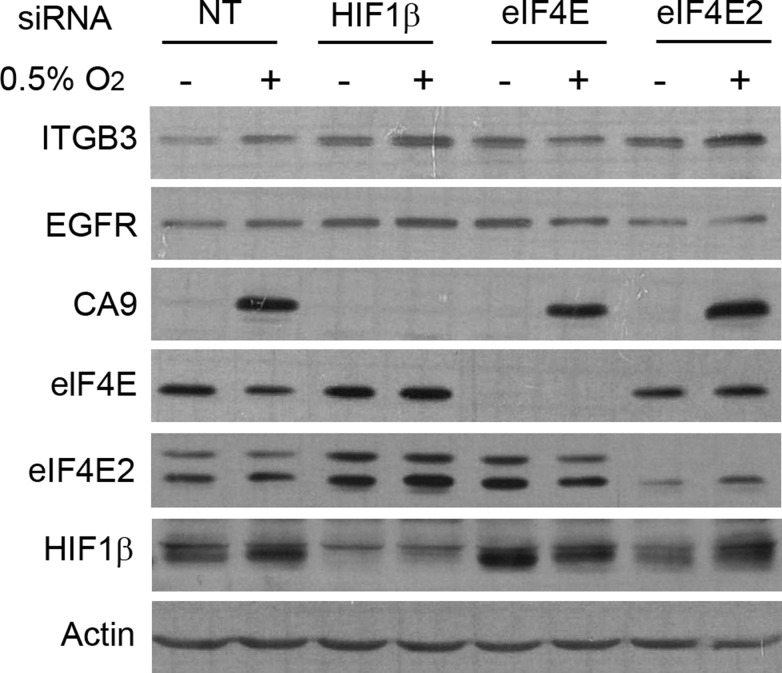
eIF4E is essential for enhanced protein synthesis of ITGB3 under low-oxygen conditions Immunoblot showing ITGB3 expression under hypoxic conditions after eIF4E2, eIF4E or HIF1β silencing in the MDA-MB-231 cell line.

Finally, we wanted to know how translation activation of ITGB3 is regulated in hypoxia. Most transcripts translated under low-oxygen conditions use the eIF4F^H^ initiation complex, especially those transcribed by the HIF transcription factor [25]. The eIF4F^H^ complex is composed of the initiation factor 4E2 (eIF4E2), eIF4A and eIF4G3. We used siRNA targeting eIF4E2 to block this mechanism of translation in hypoxic conditions. In addition, we used siRNA against HIF1β (ARNT1) to inhibit the HIF1α–HIF1β complex in charge of the transcription of HIF target genes. We also treated cells with siRNA targeting eIF4E to inhibit the standard cap-dependent translation initiation complex eIF4F. In MDA-MB-231 cells, we found that ITGB3 was still activated under low-oxygen conditions when both ARNT1 and eIF4E2 were silenced, suggesting that ITGB3 is not a HIF target gene and is not translated through the eIF4F^H^ complex. As controls, expression of CA9 and EGFR were assessed, with the findings showing that CA9 was not expressed when ARNT1 was silenced and that EGFR translation was not activated with EIF4E2 siRNA, as reported [25]. However, eIF4E inhibition blocked any induction of ITGB3 protein by hypoxia, indicating that synthesis of this integrin is cap-dependent and dependent on eIF4E and the canonical translation pathway (Figure 8). The same situation was observed in MCF10A cells (Supplementary Figure 10).

## DISCUSSION

In addition to transcription, both protein stability and translation efficiency are important determinants of the protein concentration in cells, and several screens analysing and quantifying mRNA bound to ribosomes, either by ribosome or polysome profiling, have been reported [25, 31, 35–37, 55, 56]. We measured the changes in global translation in cells subjected to hypoxia or to hypoxia plus an mTOR inhibitor, comparing non-tumoural versus tumoural human breast cancer cell lines. Although the use of polysomal fractionation combined with microarray technology has been used to identify transcripts upregulated at the protein synthesis level in tumour cells in response to hypoxia [24, 33, 34, 57, 58], our experiments allowed a comparison between non-tumoural and malignant cells using RNA-Seq technology. Our data showed that hypoxia led to transcriptional changes in 70 and 253 genes and translational changes in 94 and 65 genes in tumoural (MDA-MB-231) and non-tumoural (MCF10A) cells, respectively. When cells were treated with hypoxia + PP242, we observed 158 and 1048 transcriptionally deregulated genes and 311 and 549 genes subjected to translational regulation in the MDA-MB-231 and MCF10A cells, respectively (Supplementary Table 1). This is not simply due to an additive effect because treatment with PP242 alone only deregulated 26 and 109 genes transcriptionally and 139 and 118 transcripts at the translational level in the MDA-MB-231 and MCF10A cells, respectively. Thus, the double treatment acts synergistically at both the transcriptional and translational levels.

Another conclusion that can be drawn is that non-tumoural MCF10A cells displayed significantly more transcriptional and translational changes compared with the triple-negative MDA-MB-231 cell line. Both cell lines upregulated genes related to hypoxia and glycolysis. In hypoxia, HIF-1α plays a critical role in promoting and stimulating EMT [59–62], and downstream targets of this transcription factor were transcriptionally activated in both cell lines, as expected. In particular, MCF10A cells mainly upregulated the synthesis of factors related to cell adhesion, migration, angiogenesis and EMT in general, whereas MDA-MB-231 cells did not activate transcription or translation related to these processes in hypoxia (Figures 3 and 4). This distinction suggests that the nature of the stress response, whether protective or destructive, largely depends on the cell type. A comparison of transcripts bound to polysomes clearly revealed that non-malignant cells upregulated genes related to negative regulation of cell proliferation (*P* = 2.2 × 10^−3^) and cell death (*P* = 0.01), whereas tumour cells upregulated genes related to cell migration (*P* = 4.7 × 10^−4^) and positive regulation of cell proliferation (*P* = 9.3 × 10^−5^). One plausible explanation for this difference may be that malignant cells have already activated many genes related to these processes (EMT, proliferation, migration and angiogenesis), reducing the extent of the activation of such genes in hypoxia. On the other hand, the control of mTORC1 activity in hypoxia influences the survival response but with different outcomes in normal versus cancer cells. Whereas mTORC1 inhibition reduces the survival of normal cells in hypoxia, it supports the emergence of tumour cells that are resistant to hypoxia because, in a situation of restoration of mTOR signalling, cancer cells become sensitive to hypoxia again [31, 63]. Consistent with these results, mTOR inhibition negatively regulated GO profiles related to proliferation and growth in MCF10A cells and positively regulated apoptosis GO categories, whereas angiogenesis and tumourigenic features were identified in treated MDA-MB-231 cells (Figure 3).

In terms of translation, in hypoxia, we found similar transcriptional changes to those published by Thomas *et al*. [33] and Koritzinsky *et al*. [57] but very few translational changes compared with what was reported by Lai *et al*. [58]. This difference might be due to the use of different cell lines or more technical reasons, namely, because we isolated fractions of heavy and light polysomes, from the two ribosomes to the end, whereas Lai and colleagues isolated only the heavy polysome fractions. However, validation of our experiments revealed that the same amount and almost the same genes were translationally downregulated by mTOR inhibition compared with what has been reported and that most of these transcripts contained TOP elements [36, 37] (Supplementary Figure 4). Finally, we also validated our screening by using western blotting, showing that translationally activated transcripts in HPP correlated with an increase in protein expression in this condition (Figure 4C). More transcripts were translationally upregulated in HPP, possibly due to a synergistic effect with the hypoxia treatment. In addition to determining that transcripts activated in HPP correlated with greater protein expression in HPP, we also found that they were activated in hypoxia, as occurred with our candidate target ITGB3 (Figure 4C). A possible explanation for this finding is that treatment with the mTOR inhibitor highlighted genes still bound to polysomes under low-oxygen conditions after cap-dependent translation inhibition in the RNA-Seq and that western blotting is more sensitive and more able to detect an increase in the real protein. In addition, some transcripts activated in HPP are also increased in hypoxia.

In this study, we showed that ITGB3 is translationally activated upon hypoxia and hypoxia + PP242 in cancer and non-tumourigenic breast epithelial cells and that this protein synthesis activation was dependent on eIF4E (Figures 4C, 5A and 8). ITGB3 has been reported to be recruited to the membrane and to regulate invasion in hypoxia in glioblastoma by interacting with type III EGF receptor (EGFRvIII) in a hypoxic microenvironment enriched with vitronectin [64, 65]. It is also transcriptionally increased in Caco-2 cells simultaneously treated with EGF and hypoxia [66]. ITGB3 activates EGFR signalling through SRC-FAK-AKT and thereby promotes invasion [65]. Phenotypes of ITGB3 siRNA are stronger in activated cells with EGFRvIII than under normal conditions, and hypoxia enhances the colocalization of these two factors, preventing degradation of this receptor. In addition, activation of αvβ3 is required for metastasis in a breast carcinoma model by promoting migration *in vitro* and colonization in metastasis assays [67–71]. By inhibiting ITGB3 expression in cancer cells via infection with viral shRNA, we observed reduced migration, as expected, and increased apoptosis, but these effects were surprisingly more significant in hypoxia (Figure 5). In addition, silenced MDA-MB-231 cells showed fewer and smaller lung metastases in an *in vivo* mouse model, consistent with previous results [71] showing that downregulation of ITGB3 impairs spontaneous metastasis but not growth of the primary tumour and that ITGB3 is required by the tumour cell and not by the stroma surrounding the tumour. Integrins couple several growth factor receptors to regulate angiogenesis, survival and EMT. We found, as described previously [54], that ITGB3 is activated by the TGF-β pathway (Figure 7). ITGB3 in turn enhances TGF-β signalling by interacting physically with TGF-β receptor (TbetaR) type II via Src-mediated phosphorylation of the receptor in mammary epithelial cells [53]. TGF-β is involved in cancer cell invasion and migration through its participation in EMT. We observed that ITGB3 silencing blocked the effects of TGF-β treatment, particularly under low-oxygen conditions, especially the transcription and expression of EMT markers such as SNAIL and VIM (Figure 7). The effects of ITGB3 silencing on Smad2 phosphorylation were already visible 1 hour after TGF-β treatment and Smad2 was not phosphorylated in hypoxia when ITGB3 was silenced (Figure 7). This fits with the published data showing that the interaction between these two signalling pathways is at the receptor level, but also provides novel evidence that this pathway is particularly important under low-oxygen conditions, where ITGB3 is suggested to be more necessary [53]. This finding is consistent with the previously reported function of ITGB3 in EMT, where overexpression of ITGB3 increases motility and N-cadherin expression through binding of the FGF1 receptor and knockdown of ITGB3 suppresses the enhancement by FGF1 of TGF-β1-induced EMT in MCF10A cells [54]. Additionally, ITGB3 silencing has been reported to decrease MMP2 and MMP9 expression and reduce invasion [65, 72]. ITGB3 also increases bone metastasis [67, 73–75]. Finally, our results are consistent with data showing that genetic interference and pharmacological targeting of αv integrin (the partner of ITGB3) with the non-peptide RGD antagonist GLPG0187 in different breast cancer cell lines inhibits invasion and metastasis in the zebrafish or in a mouse xenograft model. Depletion of αv integrin in MDA-MB-231 cells also inhibits the expression of mesenchymal markers and the TGF-β/Smad response [76].

ITGB3 inhibitors have shown only modest efficacy in patients with advanced solid tumours and in tumour models *in vivo*. In this respect, our results suggest that highly hypoxic tumours may be more responsive to ITGB3 therapy. Cilengitide is an antagonist of integrins and preliminary but promising results have suggested that the microenvironment plays a role in glioblastoma progression and that ITGB3 inhibitors can act in a neoadjuvant setting to prevent metastasis rather than reduce tumours once formed [65]. Recently, other studies have used drugs to target integrin αvβ3 in glioblastoma and more recently in lung cancer [77, 78].

Finally, we demonstrated that translational activation of ITGB3 in hypoxia and hypoxia + PP242 was eIF4E dependent. During hypoxia, mTOR signalling is inhibited and translation of hypoxia-responsive genes requires alternative mechanisms, such as IRES elements [23, 79] and uORFs [80]. It is unlikely that ITGB3 is translated through an IRES-mediated mechanism because its 5′UTR is too short (21 nucleotides). Translation through HIF2a-RBM4-eIF4F^H^, a complex preferentially chosen by HIF target genes [25, 26], was also not implicated, because silencing of eIF4E2 (which forms part of eIF4F^H^) or HIF1β did not prevent activation of ITGB3 in hypoxia (Figure 8). Ho *et al*. [25] classified mRNAs into three classes depending on Te, with class III mRNAs, representing 15% of the translatome (i.e., EGFR, IGF1R), showing maintained or increased translation in hypoxia and many of the HIF targets genes belonging to this class. In contrast to that study, we found that CA9 expression was independent of eIF4E2, suggesting that the eIF4F^H^ complex is cell line dependent. Finally, we cannot discard translational regulation by microRNAs. Integrins have been shown to be downregulated by microRNAs in several studies in different types of cancer, some of which regulate ITGB3 translation, such as miR-128, which is upregulated in hypoxia [81, 82], miR-98 in hypoxia and miR-338, which inhibits migration by targeting HIF1α under low-oxygen conditions [83]. Some examples in the literature are reported for other integrins, such as TGF-β, which acts through miR-130b to increase integrin alpha 5 expression and promote migration [84]. Several miRNAs act on ITGB3 and it is possible that one of them is expressed at a low level in hypoxia and triggers an increase in the protein under these conditions.

In summary, our findings clearly show that, under hypoxic conditions, there is a clear regulation of protein synthesis in tumourigenic and non-tumourigenic cells. ITGB3 displays enhanced translational activity in hypoxia or in hypoxia combined with mTOR inhibition. ITGB3 silencing reduced cell migration and metastasis formation, most likely by partly blocking TGF-β pathway signalling. ITGB3 seems to play a particularly important role under hypoxic conditions and targeting of this integrin in such scenarios may provide an added therapeutic benefit.

## MATERIALS AND METHODS

### Cell culture and reagents

Breast cancer and non-tumourigenic cell lines were purchased from the American Type Culture Collection (ATCC) and they were authenticated by DNA profiling using short tandem repeat (STR) (GenePrint^®^ 10 System, Promega) at Genomics Core Facility, Instituto de Investigaciones Biomédicas “Alberto Sols” CSIC-UAM. MDA-MB-231, MCF7, MDA-MB-468 and BT-549 cells were maintained in Dulbecco’s modified Eagle’s medium (DMEM) (Invitrogen) supplemented with 10% heat-inactivated foetal bovine serum (FBS) (Life Technologies) and antibiotics (100 U/mL penicillin, 100 μg/mL streptomycin) (Life Technologies). MCF10A cells were maintained in DMEM supplemented with 10% FBS, 20 ng/mL EGF (#E9644; Sigma), 0.5 μg/mL hydrocortisone, 100 ng/mL cholera toxin (#C9903; Sigma) and 10 μg/mL insulin (#I9278; Sigma). Cells were maintained at 37°C in a 5% CO2 humidified incubator. To establish hypoxic conditions, cells were subjected to 0.5% O2 in 5% CO2/95% N2 and 100% humidity for 24 hours in a hypoxic chamber (*INVIVO*2 200; Ruskinn Technology, UK).

PP242 was purchased from Selleckchem (#S2218), reconstituted in dimethyl sulfoxide (DMSO) and used at a final concentration of 2.5 μM. Actinomycin D and TGF-β were purchased from Sigma-Aldrich and used at final concentrations of 5 μg/mL and 5 ng/mL, respectively. Cycloheximide was obtained from Sigma. Control cells were treated with the same amount of DMSO (vehicle).

### Sucrose density gradient fractionation and polysome and total RNA purification

Polysomal mRNA was obtained by 10%–50% sucrose gradient sedimentation. Upon hypoxia or normoxia, with and without PP242 treatments, cells were washed twice with cold 1× phosphate-buffered saline (PBS) and lysed by incubation for 10 minutes on ice in polysome buffer: 1.5 mM KCl, 5 mM Tris-HCl pH 7.4, 2.5 mM MgCl_2_, 1% Triton X-100, 1% Na-deoxycholate, 100 μg/ml cycloheximide, 2.5 μl/mL RNAaseOut and 1× Complete Roche Protease Inhibitor. The cell lysate was centrifuged at 12,000 × *g* for 15 minutes at 4°C. One microgram of total protein from the supernatant was loaded onto a 10%–50% sucrose gradient, made with the BioComp Gradient Maker, and ultracentrifuged at 37,000 rpm (SW40 rotor) for 150 minutes at 4°C. The sucrose gradient was fractionated with the ISCP UV gradient fractionation system (BioComp), connected to a UV detector to monitor absorbance at 254 nm, and the polysome profile was recorded. Twelve fractions of 900 μl each were isolated and RNA was extracted using phenol:chloroform and ethanol precipitation followed by an RNeasy Mini Kit (Qiagen) for DNase treatment according to the manufacturer’s instructions. Both total RNA and polysome-bound mRNA were analysed on an Agilent Bioanalyzer to assess RNA integrity.

### cDNA library construction, RNA sequencing (RNA-Seq) and data analysis

Total RNA was assayed for quantity and quality using Qubit^®^ RNA HS Assay (Life Technologies) and RNA 6000 Nano Assay on a Bioanalyzer 2100 (Agilent).

The RNASeq libraries were prepared from total RNA using the TruSeq™ RNA Sample Prep Kit v2 (Illumina Inc.,). Briefly, after poly-A based mRNA enrichment with oligo-dT magnetic beads from 0.5μg of total RNA as the input material, the mRNA was fragmented (resulting RNA fragment size was 80–250 nt, with the major peak at 130 nt). After first and second strand cDNA synthesis the double stranded cDNA was end-repaired, 3´adenylated and the Illumina barcoded adapters were ligated. The ligation product was enriched by 15 cycles of PCR.

The libraries were sequenced on HiSeq2000 (Illumina, Inc) in paired-end mode with a read length of 2 × 76 bp using the TruSeq SBS Kit v3. We generated in a mean of 37 million paired-end reads per sample, following the manufacturer’s protocol. Image analysis, base calling and quality scoring of the run were processed using the manufacturer’s software Real Time Analysis (RTA 1.13.48, HCS 1.5.15.1) and followed by generation of FASTQ sequence files by CASAVA.

The mRNA populations of each sample were converted to cDNA libraries using the TruSeq protocol and then sequenced using a HiSeq 2000 machine. Paired-end reads were mapped against the reference human genome (GRCh38) with STAR v2.5.1b (ENCODE parameters for long RNA) and GENCODE v24 annotation. Gene quantification was performed using RSEM v1.2.28 with default parameters. Only protein-coding genes were included in the analysis. Normalization of the count matrix was performed with the TMM method of the edgeR R package. Polysomal RNA (P) and RNA total (T) fold changes across conditions were calculated with edgeR. Significant genes (FDR < 5% for MCF10A cells and FDR < 10% for MDA-MB-231 cells) in polysomes were selected for translational efficiency calculation (log_2_FC RNA polysomes/log_2_FC RNA total). Genes with a *z*-score > 1.5 were considered to have an increased translational efficiency and genes with a *z*-score < −1.5 were considered to have a decreased translational efficiency. GO enrichment analysis of significant genes was performed with the DAVID database.

The data discussed in this publication have been deposited in NCBI’s Gene Expression Omnibus [85] and are accessible through GEO Series accession number GSE104193 (https://www.ncbi.nlm.nih.gov/geo/query/acc.cgi?acc=GSE104193).

### qRT-PCR and NanoString

One microgram of total RNA was used to synthesize cDNA using SuperScript III Reverse Transcriptase (Life Technologies). qRT-PCR was performed on a Veriti 96-well Thermal Cycler (Applied Biosystems) using SYBR Green Technology (Applied Biosystems).

**Table:** 

Gene	Forward 5′-3′	Reverse 5′-3′
ITGB3	CATCACCATCCACGACCGAA	GTGCCCCGGTACGTGATATT
SNAIL	CACTATGCCGCGCTCTTTC	GCTGGAAGGTAAACTCT GGATTAGA
VIM	CGCCAGATGCGTGAAATGG	ACCAGAGGGAGTGAATCCAGA
ECADH	CCCTCGACACCCGATTCAAA	TGGATTCCAGAAACGGAGGC
NCADH	TGTTTGACTATGAACGCAGTGG	TCAGTCATCACCTCCACCAT
YBX1	TCGCCAAAGACAGCCTAGAGA	TCTGCGTCGGTAATTGAAGTTG
HIFa	GAACGTCGAAAAGAAAAGTCTCG	CTTTATCAAGATGCGAACTCACA
Myc	TCAAGAGGCGAACACACAAC	GGCCTTTTCATTGTTTTCCA
MXD1	AGAAGTTGAAGGGGCTGGTG	TCGCTGAAGCTGGTCGATTT
CCND3	TGCACATGATTTCCTGGCCT	CTGTAGCACAGAGGGCCAAA
GAPDH	TGCACCACCAACTGCTTAGC	GGCATGGACTGTGGTCATGAG
CCDN1	AGTGGAAACCATCCGCCG	TCTGTTCCTCGCAGACCTCCA
MXI1	GGGTCCTCAGGAGATGGAAC	TGGGAGAACTCTGTGCTTTCA
FLCN	AGAGTCCTCCTCTCTCTCAGG	GGTCCACGTCTCTGCTTTTC
FNIP1	CGCCTCTTTCTTTGCAGTTCA	GGTAGCTGCTGGCACAACTT
Arrdc3	GCCCTTCAAGGACCACTGTT	AGGGGCAGGATGGTCTATCA
IRF9	GCTCTTCAGAACCGCCTACTT	CCAGCAAGTATCGGGCAAAG
ADIRF	TTGCAGGACCTGAAGCAACA	TGGTTTCCTGGGTGGTCTTG
PTGS2	AGATCATAAGCGAGGGCCAG	GGCGCAGTTTACGCTGTCTA
DLL1	CGTGGGGAGAAAGTGTGCAA	CTCTGCACTTGCATTCCCCT
BMP2	ATGGATTCGTGGTGGAGTG	GTGGAGTTCAGATGATCAGC
Actin	GCAAAGACCTGTACGCCAAC	AGTACTTGCGCTCAGGAGGA

### Knockdown analysis using siRNA transfection

Small interfering RNA (siRNA) of HOXB3, JAG2, MXI1, PTGFR, RORA, ITGB3, IRF9, EGFP (esiRNA; Sigma), eIF4E, ARNT and eIF4E2 (Dharmacon Research, Inc.) were transfected at a final concentration of 10 nM using RNAi MAX Lipofectamine (Invitrogen). After 48 hours of transfection, cells were seeded for MTT assays, migration assays or western blot analysis.

### Modulation of expression using retroviral and lentiviral infection

For lentiviral shRNA of ITGB3, pLKO.1-puro-shITGB3 was constructed by annealing the oligonucleotides 5′-ccgggccaagactcatatagcattgctcga-3′ and 5′-aattcaaaaagccaagactcatatagcatt-3′ and cloning them into a pLKO1 vector. The shITGB3 #3 was obtained from Dharmacon. We also used pLKO1 as shCTL. MGC Human ITGB3 Sequence-Verified cDNA (CloneId: 40128462) was obtained from Dharmacon and was subcloned into pLPCX retroviral plasmid after annealing of the oligonucleotides 5′-CTTAGATCTA CCATGCGAGC GCGGCCGCGG CCC-3′ and 5′-GGTAAGCTTT TAAGTGCCCC GGT ACGTGAT ATT-3′. Production of lentiviruses and retroviruses and their infection of target cells were performed as previously described [86]. Infected MDA-MB-231 and MCF10A cells were selected with 0.7 μg/mL or 1.5 μg/mL puromycin for 3–4 days, respectively. Viral production and infection were performed at 37°C. All of these plasmids were sequenced twice from both ends to ensure expression of the correct coding sequence.

### MTT assays

MTT (3-[4,5-dimethylthiazol-25-yl]-2.5-dipheny ltetrazolium bromide; Sigma) was added to the medium to a final concentration of 0.5 mg/mL and incubated for 4 hours at 37°C. The medium was then removed and 0.2 mL DMSO was added. Absorbance was measured at 590 nm by using a Synergy spectrophotometer (Biotek). Readings were taken 0, 24, 48, 72 and 96 hours after cell treatment.

### Migration assays

Cells were plated in 24-well plates in triplicate. After 24 hours, the cells were treated overnight with mitomycin C (5 μg/mL, Santa Cruz Biotechnology). Then, a wound was made in the monolayer with a pipette tip, the medium was replaced and the cells were incubated under normoxic or hypoxic conditions. Pictures of the wounds were taken 0, 8 and 24 hours after treatment initiation, and wound closure was measured using ImageJ software.

### Caspase assays

To measure caspase-3 and -7 activity, the Caspase-Glo 3/7 Assay (Promega) was used. Five thousand cells in 200 μL were seeded in black-walled 96-well plates and subjected to normoxia or hypoxia for 48 hours. Then, 100 μL Caspase-Glo 3/7 reagent was added to each well and the cells were incubated at room temperature for 1 hour. Luminescence was measured using a Synergy Mx Monochromator-Based Multi-Mode Microplate Reader.

### Protein extraction and immunoblotting

Total protein extracts were generated using lysis buffer (50 mM Tris-HCl, pH 7.4, 150 mM NaCl, 1% Triton X-100, 1% sodium deoxycholate, 0.1% SDS, 1 mM EDTA) supplemented with PhosSTOP and Complete Phosphatase/Protease Inhibitor Cocktails (Roche Diagnostics GmbH, Mannheim, Germany). Protein extracts (20–25 μg per sample) were loaded onto SDS-PAGE gels and transferred electrophoretically to PVDF membranes and immunodetection of proteins was performed using ECL™ Western Blotting Detection Reagents (GE Healthcare, Buckinghamshire, UK). The following primary antibodies were used: anti-Myc, anti-HIF1a, anti-4EBP1, anti-eIF4E, anti-YBX1, anti-Snail, anti-CA9, anti-Smad2/3, anti-β-catenin (Cell Signaling), anti-phospho Smad2 (Millipore), anti-β3 integrin, anti-MMP3, anti-N-cadherin (Abcam), anti-CycD1, anti-vimentin (Santa Cruz Biotechnology), anti-BMP2, anti-CCDC103, anti-TTC30B, anti-EIF3G, anti-RPL11 (CusaBio), anti-Cx31 (Alpha Diagnostics), anti-eIF4E2 (GeneTex), anti-αv integrin and anti-β-actin (1:500; Calbiochem, Darmstadt, Germany). Anti-mouse and anti-rabbit HRP secondary antibodies were from Pierce. Bound antibodies were visualized with an enhanced chemiluminescence detection kit (Amersham Pharma-Biotech).

### Animal study

Female athymic nude mice (Harlan Interfauna Iberica, Barcelona, Spain) were kept in pathogen-free conditions and used at 7 weeks of age. Animal care was handled in accordance with the Guide for the Care and Use of Laboratory Animals of the Vall d’Hebron University Hospital Animal Facility, and the experimental procedures were approved by the Animal Experimentation Ethical Committee at the institution. All of the *in vivo* studies were performed by the ICTS ‘NANBIOSIS’, more specifically at the CIBER-BBN *in vivo* Experimental Platform of the Functional Validation & Preclinical Research (FVPR) area (http://www.nanbiosis.es/portfolio/u20-in-vivo-experimental-platform/) (Barcelona, Spain).

Mice received an intravenous injection of tumour cells (MDA-MB-231 parental cells or MDA.MB-231-shITGB3, 2 × 10^6^ cells per inoculum) into the left caudal tail vein. Animals’ body weight and physical appearance were measured twice a week. Two set of experiments were performed. To record animal survival after inoculation, animals were kept alive until they met the established ethical criteria (in accordance with the protocol approved by the Experimental Animal Ethics Committee). In the second set of experiments, all animals were euthanized 36 days after inoculation to compare the number and extent of the lung metastases between groups. In these experiments, the lungs were collected, weighed and fixed with Bouin’s solution. The number and size of lung metastases were quantified macroscopically using a stereoscopic microscope. Lungs were processed later for histopathological analyses.

### Statistics

Results are expressed as means + standard errors of the means. The two-tailed Student’s *t*-test was used for statistical analysis. *P* < 0.05 was considered significant. To analyse the distributions of qualitative variables, the Pearson coefficient was used.

## SUPPLEMENTARY MATERIALS FIGURES AND TABLE





## Abbreviations

CTL: Control
EGFR: Epidermal growth factor receptor
eIF4F: Eukaryotic initiation factor 4F
EMT: epithelial to mesenchymal transition
GO: Gene Onthology
H: Hypoxia
HER2: human epidermal growth factor receptor type 2
HIF1α: hypoxia-inducible factor 1
HPP: Hypoxia + PP242
IRES: Internal Ribosome Entry Site
ITGB3: Integrin Beta 3
mTOR: mechanistic target of rapamycin
N: Normoxia
NPP: Normoxia + PP242
PAM50: Prediction Analysis of Microarray 50
PCA: principal component analysis
qRT-PCR: Quantitative reverse transcription PCR
RNA-Seq: high-throughput RNA sequencing
shRNA: short interfering RNA
siRNA: short hairpin RNA
TGF-β: Transforming growth factor beta
TNBC: Triple-negative breast cancer
UTR: untranslated region
