# Supplementary material for: Hypoxia-mediated translational activation of ITGB3 in breast cancer cells enhances TGF-β signaling and malignant features in vitro and in vivo
Source: Oncotarget. 2017 Dec 12;8(70):114856–76. doi: 10.18632/oncotarget.23145 (PMC5777738; doi:10.18632/oncotarget.23145)
Supplement: Supplementary file 1 [file oncotarget-08-114856-s001.pdf]

Hypoxia-mediated translational activation of ITGB3 in breast cancer cells enhances TGF-β signaling and malignant features *in vitro* and *in vivo*

SUPPLEMENTARY MATERIALS

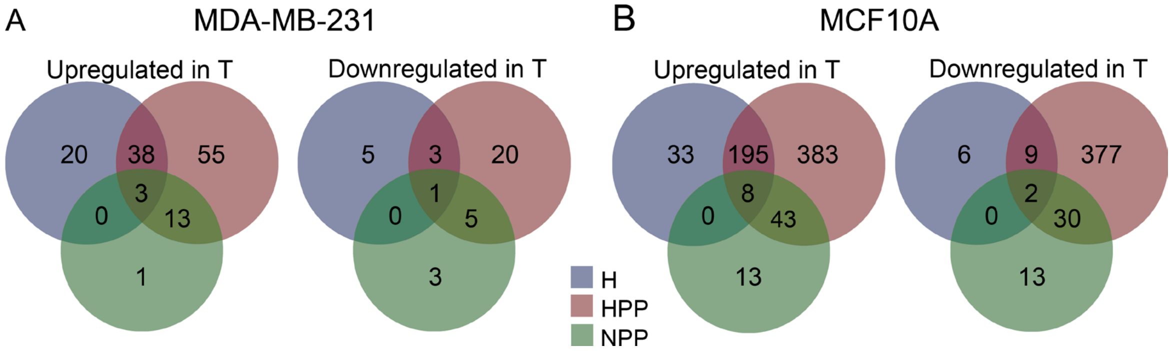

Supplementary Figure 1: Venn diagrams showing the distribution of transcriptionally deregulated genes in H, HPP and NPP in MDA-MB-231 (**A**) and MCF10A (**B**) cell lines.

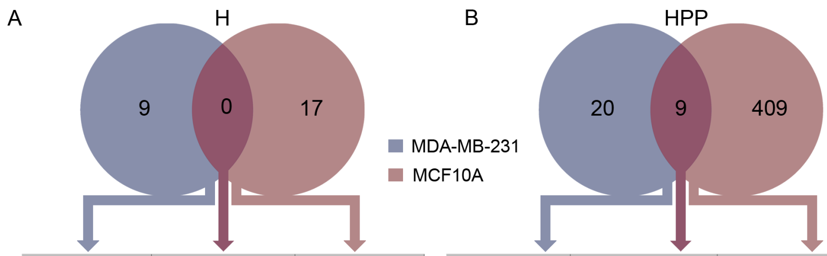

A

ONLY in MDA-MB-231	Intersection MDA-MB-231 and MCF10A	ONLY in MCF10A
embryonic hemopoiesis		embryonic digestive tract development
**positive regulation of cell proliferation**		eye development
visual learning		**negative regulation of cell proliferation**
muscle contraction		**intracellular signal transduction**
peptidyl-tyrosine phosphorylation		**Pathways in cancer**
signal transduction		Malaria
**positive regulation of gene expression**		
**Pathways in cancer**		
Central carbon metabolism in cancer		

B

ONLY in MDA-MB-231	Intersection MDA-MB-231 and MCF10A	ONLY in MCF10A
O-glycan processing	peptidyl-tyrosine dephosphorylation	**negative regulation of transcription from RNA polymerase II promoter**
	**G1/S transition of mitotic cell cycle**	**negative regulation of transcription, DNA-templated**
		transcription, DNA-templated
		**positive regulation of transcription from RNA polymerase II promoter**
		cell maturation
		odontogenesis of dentin-containing tooth
		**G1/S transition of mitotic cell cycle**
		protein localization to nucleus
		DNA replication initiation
		RNA phosphodiester bond hydrolysis, endonucleolytic
		membranous septum morphogenesis
		**BMP signaling pathway**
		positive regulation of transcription, DNA-templated
		DNA replication
		positive regulation of osteoblast differentiation
		covalent chromatin modification
		**positive regulation of cell death**
		embryonic cranial skeleton morphogenesis
		regulation of DNA replication
		T cell receptor V(D)J recombination
		regulation of transcription from RNA polymerase II promoter
		transcription from RNA polymerase II promoter
		macrophage differentiation
		**cell division**
		anterior/posterior pattern specification
		**negative regulation of cell proliferation**
		generation of neurons
		pharyngeal arch artery morphogenesis
		kidney development
		cellular respiration
		**regulation of transcription involved in G1/S transition of mitotic cell cycle**
		outflow tract septum morphogenesis
		negative regulation of BMP signaling pathway
		neuron differentiation
		DNA methylation
		**Wnt signaling pathway**
		negative regulation of pathway-restricted SMAD protein phosphorylation
		**regulation of epithelial cell proliferation**
		organ induction
		regulation of transcription, DNA-templated
		embryonic forelimb morphogenesis
		**canonical Wnt signaling pathway**
		cellular response to lithium ion
		aorta morphogenesis
		lung morphogenesis
		**positive regulation of cell proliferation**
		**Hippo signaling pathway**
		**Pathways in cancer**
		Prostate cancer
		HTLV-I infection
		Basal cell carcinoma
		**TGF-beta signaling pathway**
		Small cell lung cancer
		Colorectal cancer
		Signaling pathways regulating pluripotency of stem cells
		**Cell cycle**
		Acute myeloid leukemia
		DNA replication
		Hepatitis B
		**Ribosome biogenesis in eukaryotes**
		**Transcriptional misregulation in cancer**

Supplementary Figure 2: Analysis of transcriptionally downregulated genes under hypoxic and hypoxic + PP242 conditions. (**A**) Venn diagram with the distribution of transcripts downregulated in hypoxia. A table showing the main GO categories (with $P < 0.03$) and Kegg pathways (with $P < 0.03$) associated with each cell line. (**B**) Venn diagram with the distribution of transcripts downregulated in hypoxia + PP242. A table showing the main GO categories (with $P < 0.03$) and Kegg pathways (with $P < 0.03$) associated with each cell line.

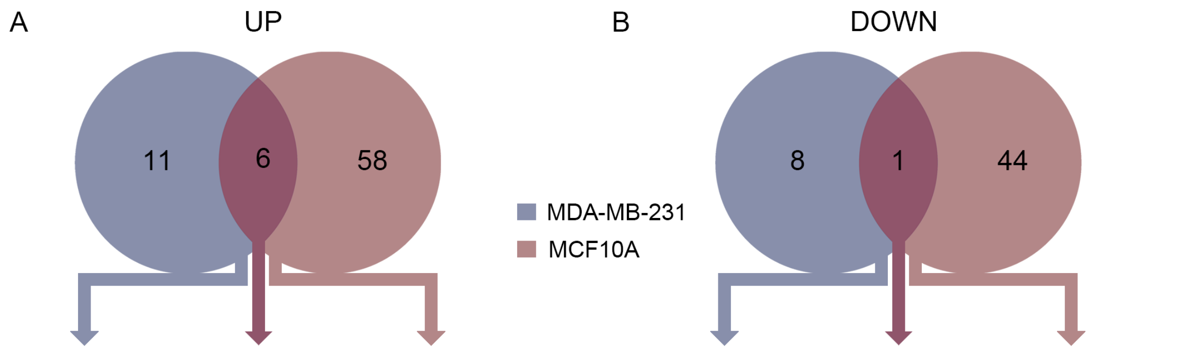

ONLY in MDA-MB-231	Intersection MDA-MB-231 and MCF10A	ONLY in MCF10A
innate immune response in mucosa	regulation of sequence-specific DNA binding transcription factor activity	**negative regulation of transcription from RNA polymerase II promoter**
antibacterial humoral response	negative regulation of cell cycle	decidualization
response to endoplasmic reticulum stress	**cell cycle**	response to estradiol
Systemic lupus erythematosus		cellular response to lipopolysaccharide
Alcoholism		**epidermal growth factor receptor signaling pathway**
		cellular response to interleukin-1
		response to endoplasmic reticulum stress
		response to metal ion
		**negative regulation of cell proliferation**
		Hematopoietic cell lineage
		**Transcriptional misregulation in cancer**

ONLY in MDA-MB-231	Intersection MDA-MB-231 and MCF10A	ONLY in MCF10A
anatomical structure formation involved in morphogenesis		**negative regulation of transcription from RNA polymerase II promoter**
phosphorylation		negative regulation of pathway-restricted SMAD protein phosphorylation
		negative regulation of oligodendrocyte differentiation
		negative regulation of sequence-specific DNA binding transcription factor activity
		lung morphogenesis
		**BMP signaling pathway**
		proximal/distal pattern formation
		pituitary gland development
		**negative regulation of Notch signaling pathway**
		negative regulation of osteoblast differentiation
		cerebral cortex GABAergic interneuron fate commitment
		**negative regulation of BMP signaling pathway**
		positive regulation of transcription from RNA polymerase II promoter
		phosphatidylinositol-3-phosphate biosynthetic process
		odontogenesis of dentin-containing tooth
		regulation of transcription from RNA polymerase II promoter involved in forebrain neuron fate commitment
		cell differentiation in hindbrain
		subpallium development
		**negative regulation of transforming growth factor beta receptor signaling pathway**
		prostatic bud formation
		negative regulation of SMAD protein complex assembly
		lung development
		anterior/posterior pattern specification
		response to laminar fluid shear stress
		**transforming growth factor beta receptor signaling pathway**
		mammary gland epithelial cell proliferation
		neuron differentiation
		positive regulation of gene expression
		**G1/S transition of mitotic cell cycle**
		membranous septum morphogenesis
		osteoblast differentiation
		transcription, DNA-templated
		negative regulation of neural precursor cell proliferation
		transcription from RNA polymerase II promoter
		**TGF-beta signaling pathway**
		**Hippo signaling pathway**
		**Pathways in cancer**
		**Signaling pathways regulating pluripotency of stem cells**
		Melanoma

Supplementary Figure 3: Analysis of transcriptionally deregulated genes when cells are treated with PP242. (**A**) Venn diagram with the distribution of transcripts upregulated in NPP. A table showing the main GO categories (with $P < 0.03$) associated with each cell line and the GO categories (with $P < 0.03$) associated with genes in the intersection between the two cell lines. (**B**) Venn diagrams with the distribution of transcripts downregulated in NPP. A table showing the main GO categories (with $P < 0.03$) associated with each cell line and the GO categories (with $P < 0.03$) associated with genes in the intersection between the two cell lines.

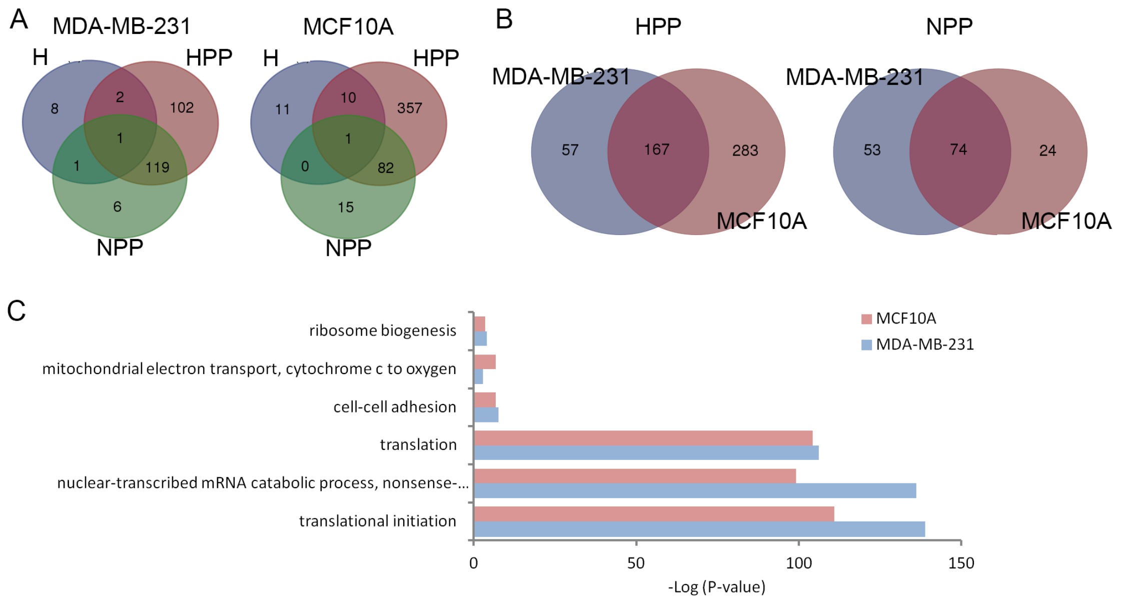

D

	Te down in MCF10A	Te down in MDA-MB-231	Te down in Hsieh et al., 2012 (INK128, PC-3 cells)	Te down in Thoreen et al., 2012 (Torin1 , MEFs)
**Te down in MCF10A**	98	74	58	26
**Te down in MDA-MB-231**		127	78	32
**Te down in Hsieh et al., 2012 (INK128, PC-3 cells)**			89	28
**Te down in Thoreen et al., 2012 (Torin1 , MEFs)**				37

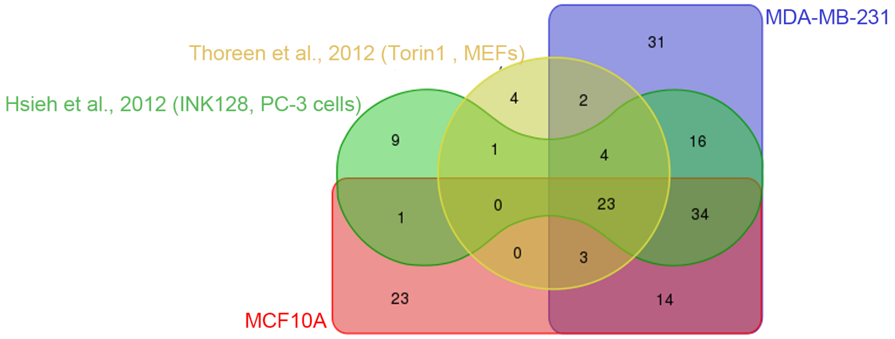

Supplementary Figure 4: Translationally downregulated transcripts upon PP242 treatment. (**A**) Venn diagrams showing the distribution of downregulated transcripts at the protein synthesis level under H, HPP and NPP conditions in each cell line. (**B**) Venn diagrams showing translationally downregulated transcripts in HPP and NPP highlighting genes in the intersection between the two cell lines. (**C**) Main GO categories associated with translationally downregulated genes in HPP in both cell lines. (**D**) Above: Table showing the number of genes translationally downregulated in our study and in others studies. Below: Venn diagram showing the number of genes translationally inactivated in NPP compared with the ones inactivated in other published studies.

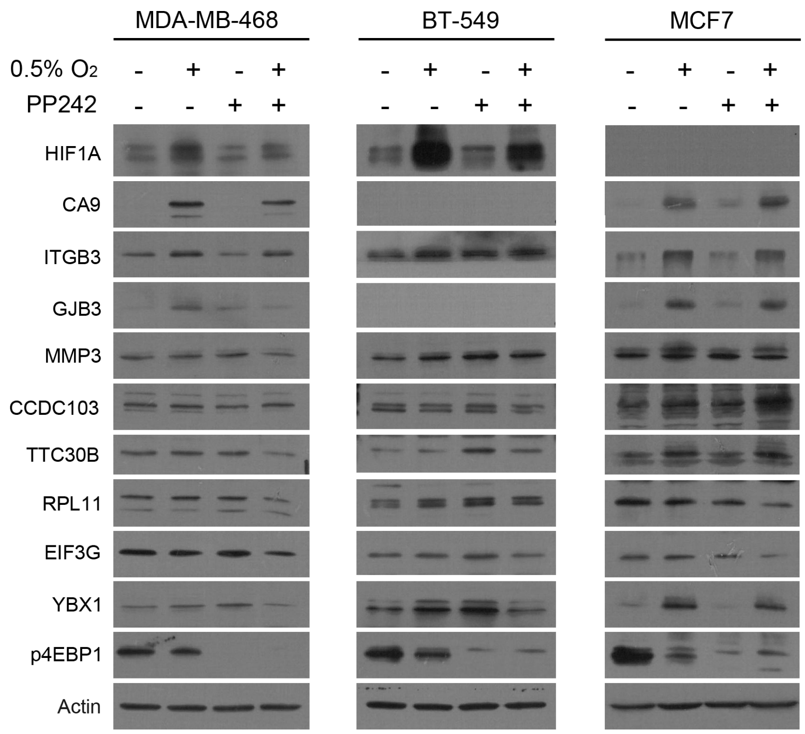

Supplementary Figure 5: Increased translational efficiency (Te) of ITGB3 is accompanied by increased protein in other breast cancer cell lines. Immunoblots for all experimental conditions of the different translationally activated (ITGB3, GJB3, MMP3, CCDC103, TTC30B) or inactivated (RPL11, EIF3G, YBX1) targets in HPP in MDA-MB-468, BT-549 and MCF7.

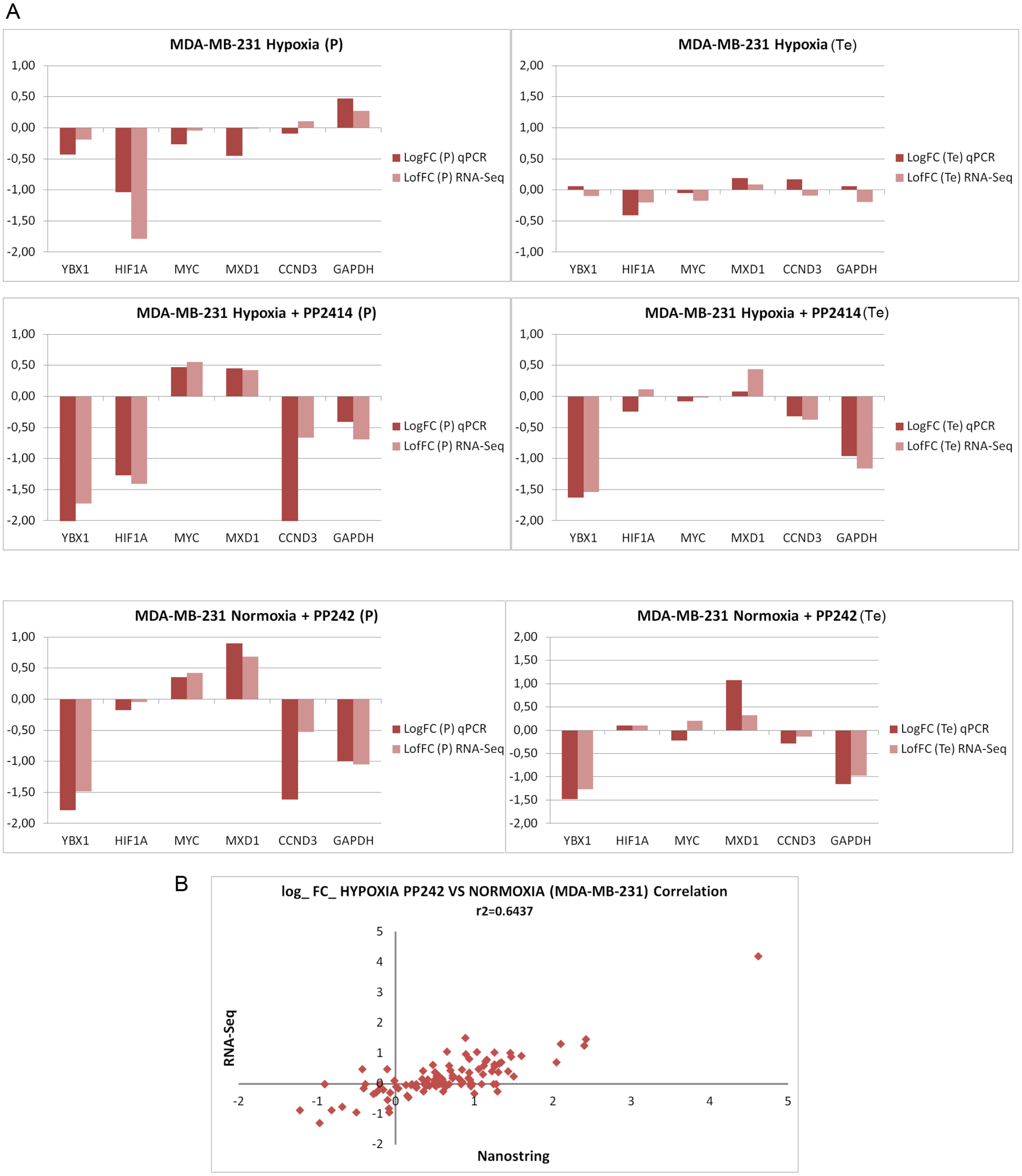

Supplementary Figure 6: Validation of the screening by qRT-PCR and NanoString in MDA-MB-231 cells. (**A**) qRT-PCR of candidate genes in polysomal mRNA (left panels) and the ratio of Log$_2$FC P to Log2FC T (right panels) compared with the values obtained from the RNA-Seq screening under H, HPP and NPP conditions. (**B**) Correlation between the 50-gene PAM50 assay and the expression obtained in RNA-Seq for these genes from the total mRNA.

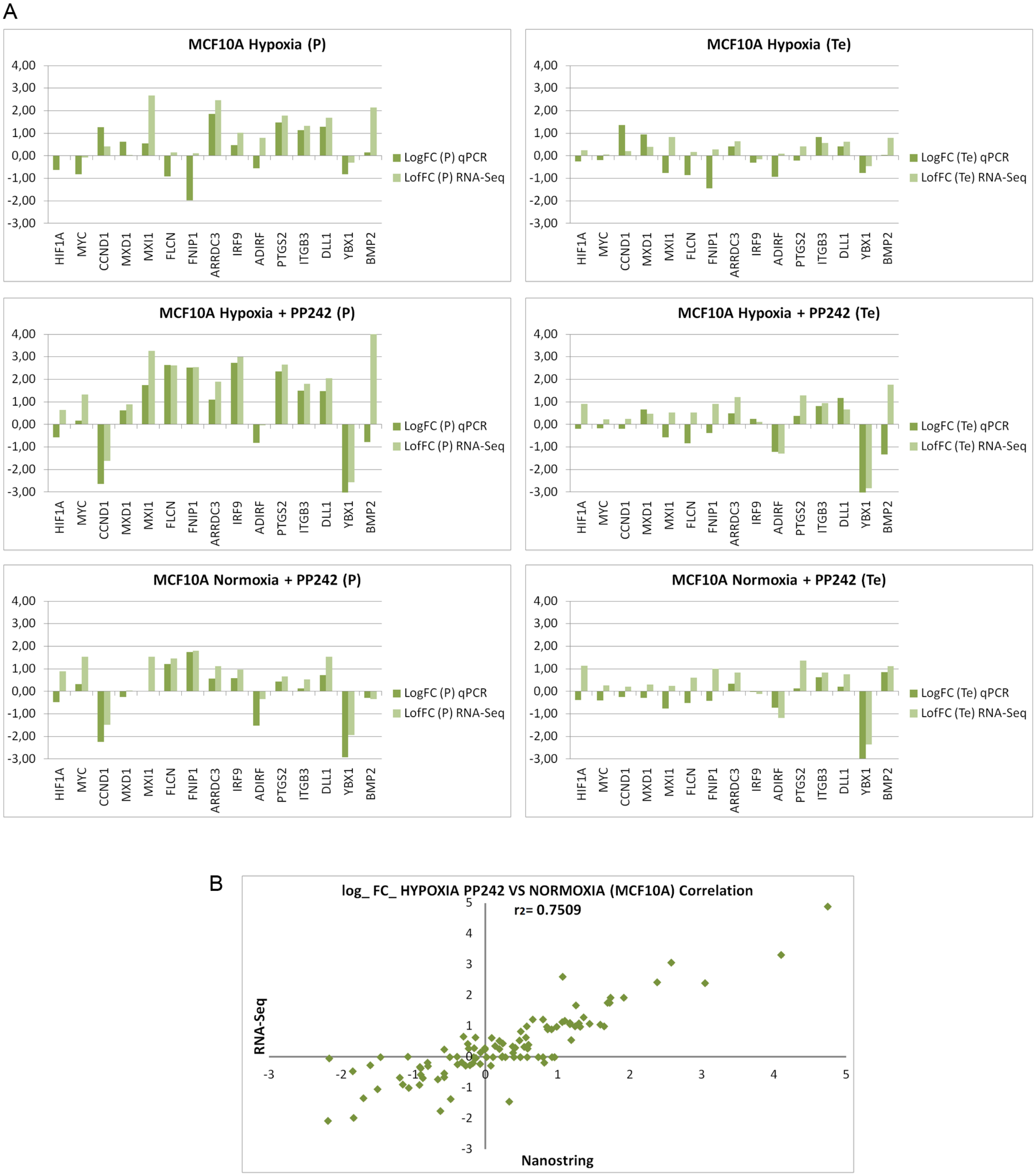

Supplementary Figure 7: Validation of the screening by qRT-PCR and NanoString in MCF10A cells. (**A**) qRT-PCR of candidate genes in polysomal mRNA (left panels) and the ratio of Log_2FC P to Log_2FC T (right panels) compared with the values obtained from the RNA-Seq screening under H, NPP and HPP conditions. (**B**) Correlation between the 50-gene PAM50 assay and the expression obtained in the RNA-Seq for these genes from the total mRNA.

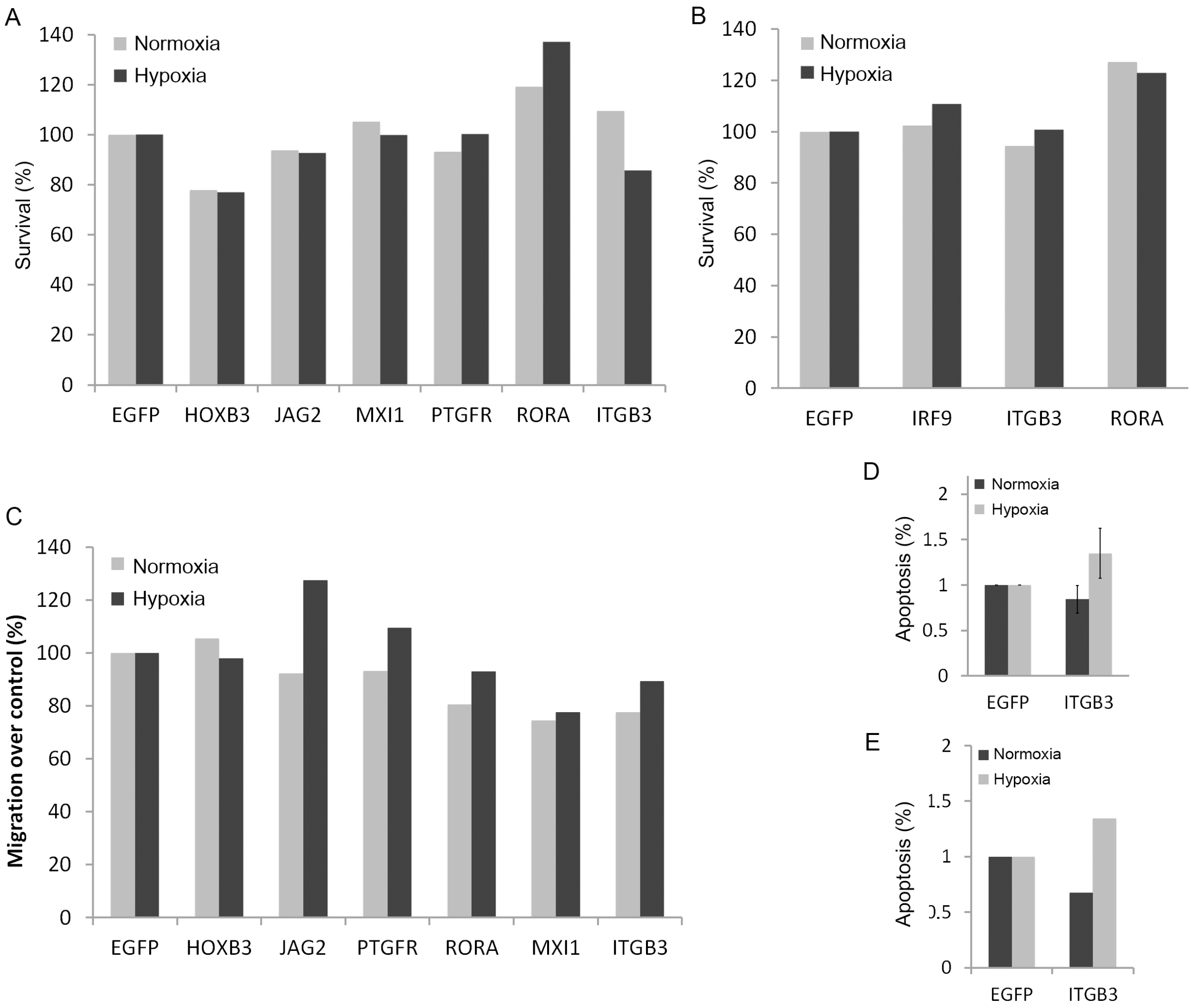

Supplementary Figure 8: Screening by siRNA of candidate translationally activated genes in hypoxia with PP242. (**A**) Forty-eight hour survival rates in hypoxia compared with normoxia. (**B**) Forty-eight hour survival rates in hypoxia compared with normoxia in MCF10A cells transfected with the different siRNAs. (**C**) Migration assays in MDA-MB-231 cells transfected with the different siRNAs and subjected to hypoxia versus normoxia for 8 hours. (**D**) Apoptosis in MDA-MB-231 cells transfected with the different siRNAs. (**E**) Apoptosis in MCF10A cells transfected with the different siRNAs.

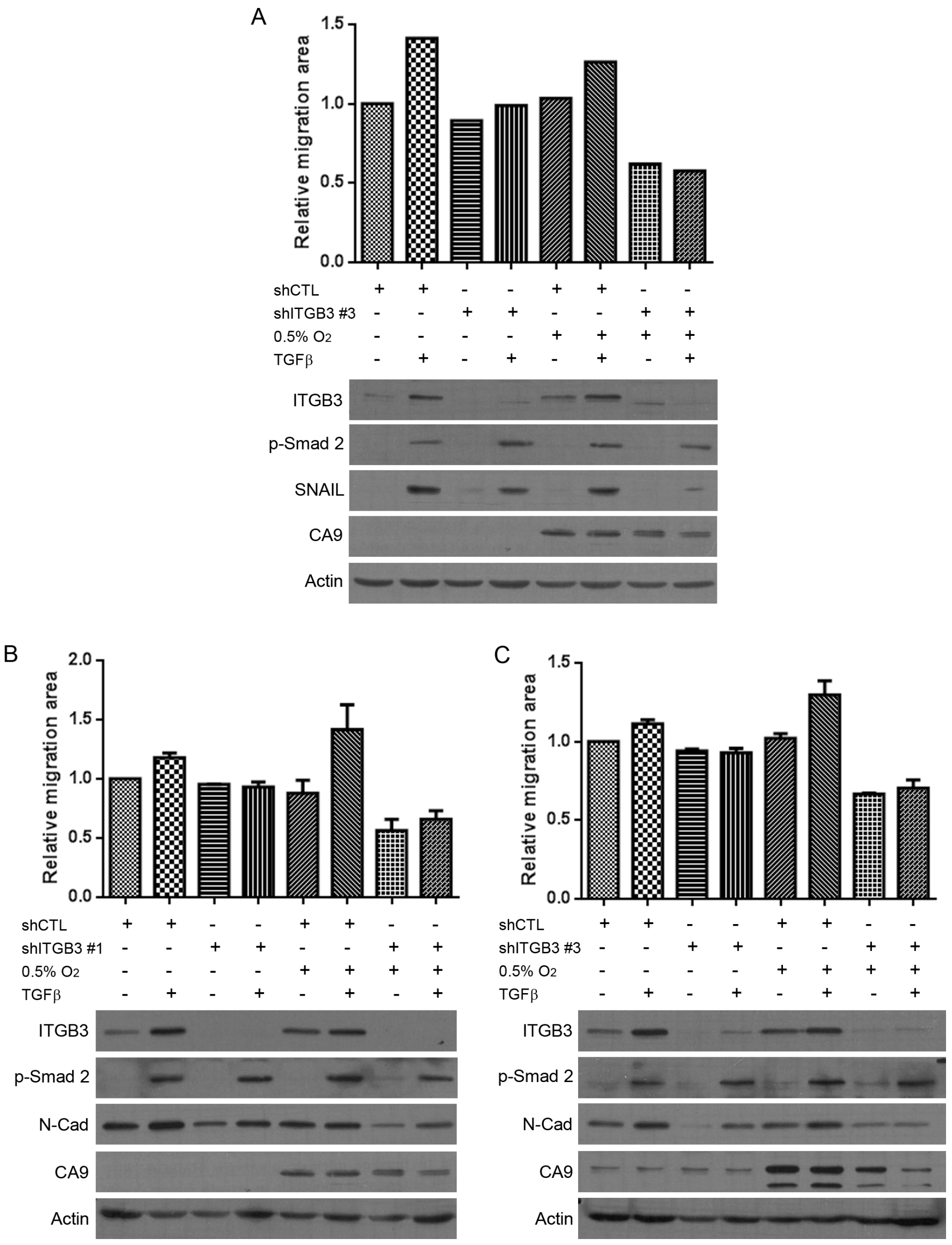

Supplementary Figure 9: Migration assay of MDA-MB-231 and MCF10A cells with ITGB3 silencing and treated with TGB-β. (**A**) Migration assay of MDA-MB-231 cells using shITGB3 #3 (above) and an immunoblot showing ITGB3 and Snail expression upon treatments (below). (**B**) Migration assay of MCF10A cells using shITGB3 #1 (above) and an immunoblot showing ITGB3 and N-cadherin expression upon treatments (below). (**C**) Migration assay of MCF10A cells using shITGB3 #3 (above) and an immunoblot showing ITGB3 and N-cadherin expression upon treatments (below).

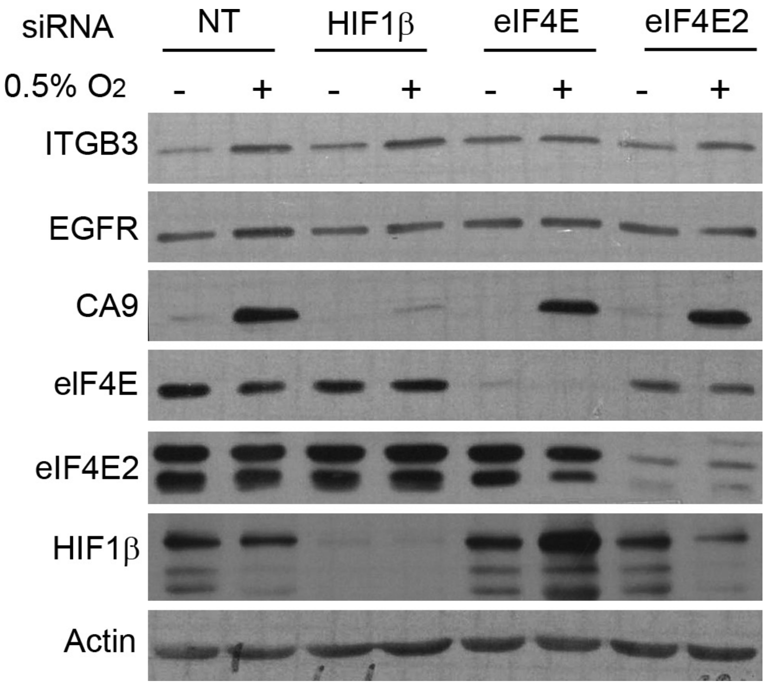

Supplementary Figure 10: eIF4E is essential for protein synthesis activation of ITGB3 under low-oxygen conditions. Immunoblot showing ITGB3 expression under hypoxic conditions after eIF4E2, eIF4E or HIF1β silencing in the MCF10A cell line.

Supplementary Table 1: Lists of mRNAs that are translationally regulated in MCF10A and MDA-MB-231 cells in all experimental conditions. See Supplementary_Table_1
